# Group B Streptococcal Maternal Colonization and Neonatal Disease: Molecular Mechanisms and Preventative Approaches

**DOI:** 10.3389/fped.2018.00027

**Published:** 2018-02-22

**Authors:** Kathryn A. Patras, Victor Nizet

**Affiliations:** ^1^Division of Host-Microbe Systems & Therapeutics, Department of Pediatrics, University of California, San Diego, La Jolla, CA, United States; ^2^Skaggs School of Pharmacy and Pharmaceutical Sciences, University of California, San Diego, La Jolla, CA, United States

**Keywords:** group B *Streptococcus*, neonatal sepsis, vaginal colonization, postpartum disease, virulence factors, intrapartum antibiotic prophylaxis

## Abstract

Group B *Streptococcus* (GBS) colonizes the gastrointestinal and vaginal epithelium of a significant percentage of healthy women, with potential for ascending intrauterine infection or transmission during parturition, creating a risk of serious disease in the vulnerable newborn. This review highlights new insights on the bacterial virulence determinants, host immune responses, and microbiome interactions that underpin GBS vaginal colonization, the proximal step in newborn infectious disease pathogenesis. From the pathogen perspective, the function GBS adhesins and biofilms, β-hemolysin/cytolysin toxin, immune resistance factors, sialic acid mimicry, and two-component transcriptional regulatory systems are reviewed. From the host standpoint, pathogen recognition, cytokine responses, and the vaginal mucosal and placental immunity to the pathogen are detailed. Finally, the rationale, efficacy, and potential unintended consequences of current universal recommended intrapartum antibiotic prophylaxis are considered, with updates on new developments toward a GBS vaccine or alternative approaches to reducing vaginal colonization.

Summary: This review provides an update on group B *Streptococcus* factors promoting maternal colonization and considerations for current and developing neonatal disease prevention strategies.

## Introduction

*Streptococcus agalactiae* [group B *Streptococcus* (GBS)] is an encapsulated Gram-positive bacterium that colonizes the lower gastrointestinal tract, and in females, the urogenital tract, of 20–30% of healthy human adults (1). GBS utilizes multiple adhesins and stress response mechanisms, defenses against other microbes, and immune evasion strategies to achieve persistent or intermittent vaginal colonization. During the peripartum period, GBS gains access to a new host, the immune-deficient neonate, where GBS can again serve as a commensal organism or transition to an invasive pathogen resulting in sepsis or meningitis. GBS displays an arsenal of virulence factors, including a potent hemolytic toxin and multiple surface proteins to invade host tissues, as well as molecular mimicry and proteases to impede host immune recognition and responses. Maternal screening and antibiotic therapy during labor are the current preventive measures against GBS neonatal disease. However, this early exposure to broad-spectrum antibiotics alters the infant gut flora and may be accompanied with life-long consequences. This review provides a collection of recent findings on the epidemiology, molecular pathogenesis and host immune responses related to GBS vaginal colonization, and an outlook on emerging alternative prophylactic strategies to limiting maternal vaginal colonization and neonatal exposure.

## Emergence of GBS as a Human Neonatal Pathogen

In the 1970s, GBS emerged as the leading cause of mortality and morbidity in human neonates, causing over 7,000 cases of invasive neonatal infections annually in the U.S. at that time (2). Early-onset GBS disease (EOD) results from ascending infection of the womb or by neonatal acquisition during vaginal passage and manifests on days 0–6 of life with pneumonia or respiratory distress commonly advancing to sepsis. Late-onset disease (LOD) is classified with onset from days 7 to 90, arising from maternal, nosocomial, or community sources, and presents with bacteremia with a high complication rate of meningitis (3, 4). Of the children developing GBS meningitis, almost 50% will have consequences of neurological disability (5). An estimated four million newborns die each year within the first 4 weeks of life globally, and one in four of these deaths stems from severe infection including sepsis or pneumonia with 99% of neonatal deaths occur in low- and middle-income countries (6). In developed countries, GBS and *E. coli* combined cause approximately 70% of early-onset neonatal sepsis of both term and preterm infants (7). Neonatal colonization occurs in approximately 40–75% of births from GBS colonized mothers, with 1–2% of cases leading to invasive disease (8–11). Risk factors for neonatal colonization include intrapartum fever and heavy maternal colonization, lack of intrapartum antibiotic prophylaxis (IAP) exposure, and also African ethnicity (12). Risk factors for neonatal infection include preterm delivery <37 weeks, prolonged rupture of membranes (>18 h), intrapartum fever temperature of at least 38°C or 100.4°F, a prior infant with GBS infection, or exposure but not infection with HIV (13, 14). Infants born to GBS-positive mothers are also three times more likely to be transferred to the neonatal intensive care unit (15). GBS exposure or colonization may also impact health later in childhood, as maternal GBS colonization has been associated with a significant increased risk of childhood asthma (16). Increasing in immune-compromised adults, including pregnant women, diabetics and the elderly, GBS is recognized as an invasive pathogen, with reports of sepsis, urinary tract infections, soft tissue infections, and meningitis (3).

## GBS Phylogeny and Host Range

Until the 1930s, GBS was considered primarily bovine in origin and recognized as a frequent etiologic agent of mastitis (17). GBS has since been readily isolated from various mammals, reptiles, and fishes, both as a commensal and pathogen (18). GBS is now a rising concern in aquaculture, particularly the tilapia industry, causing an estimated 40 million dollar in losses annually, and serving as a potential additional route of zoonotic infection (19). Little is known about the dissemination of GBS across species; however, a cross-sectional cohort study revealed that cattle exposure was a predictor of human GBS colonization indicating interspecies transmission can occur (20). Phylogenetic analysis of bovine and human invasive GBS strains suggests that hyperinvasive human neonatal isolates have recently diverged from a bovine ancestor (17). In the 1930s, Dr. Rebecca Lancefield described two polysaccharide antigens: the conserved Group B carbohydrate, and the diverse S substance that generates type-specific antisera (21). Since then, 10 variants of the capsule have been described (Ia, Ib, and II–IX), with serotypes Ia, Ib, II, III, and V most commonly isolated from humans (4, 22, 23). More recently, GBS has been classified by sequence type (ST) based on an allelic profile of seven different loci, with the majority of GBS human isolates being ST-1, ST-17, ST-19, or ST-23 (24). Diversity of the GBS polysaccharide capsule may allow for its broad range of hosts, in part through the establishment of biofilms (25).

## Epidemiology of GBS Vaginal Colonization

A broad range of GBS vaginal colonization rates during pregnancy have been reported, and this variance depends on the regions or populations of individual studies as well as the method of sampling and culturing. The most recent report of global vaginal GBS colonization estimates a prevalence of 18%, after adjusting for sample collection and methodology, with the lowest regional prevalence in Southern and Eastern Asia (11–13%) and highest prevalence in the Caribbean (35%) (26). Previous global estimates were similar, falling in the range of 8–18% (27–29). Numerous risk factors for GBS vaginal colonization have been identified both biological and socioeconomic in nature. Biological factors include a history of premature rupture of membranes (PROM) (30), gastrointestinal GBS colonization (31), and increased maternal age. In one study, maternal age >36 years of age was associated with persistent colonization (32), and another demonstrated a higher GBS colonization rate in women >40 years of age (33). Ethnicity, obesity, low vitamin D intake, hygiene, sexual activity, health care occupation, and illiteracy have also been associated with GBS vaginal carriage (31, 34–36). During pregnancy, GBS vaginal colonization may be continuous, intermittent, or transient among individual women (37). The majority of GBS-positive women are stably colonized during the peripartum period; however, changes in serotype or ST or subsequent loss of specific STs have been documented (32). Many studies have examined the most common serotypes of colonizing strains in the United States and are in agreement that serotypes Ia, III, and V are the most represented serotypes (32, 38, 39) with serotype III being more likely to result in persistent colonization than Ia or V (40). Given the possibility of capsular switching, more recent studies have determined GBS STs and found STs 1, 23, and 19 the most abundant colonizing strains (32, 41). Strong biofilm formation was recently determined to be a trait of asymptomatic colonizing strains, with weak biofilm capacity present in invasive strains (42).

## Bacterial Determinants of GBS Vaginal Colonization

**Figure 1:**
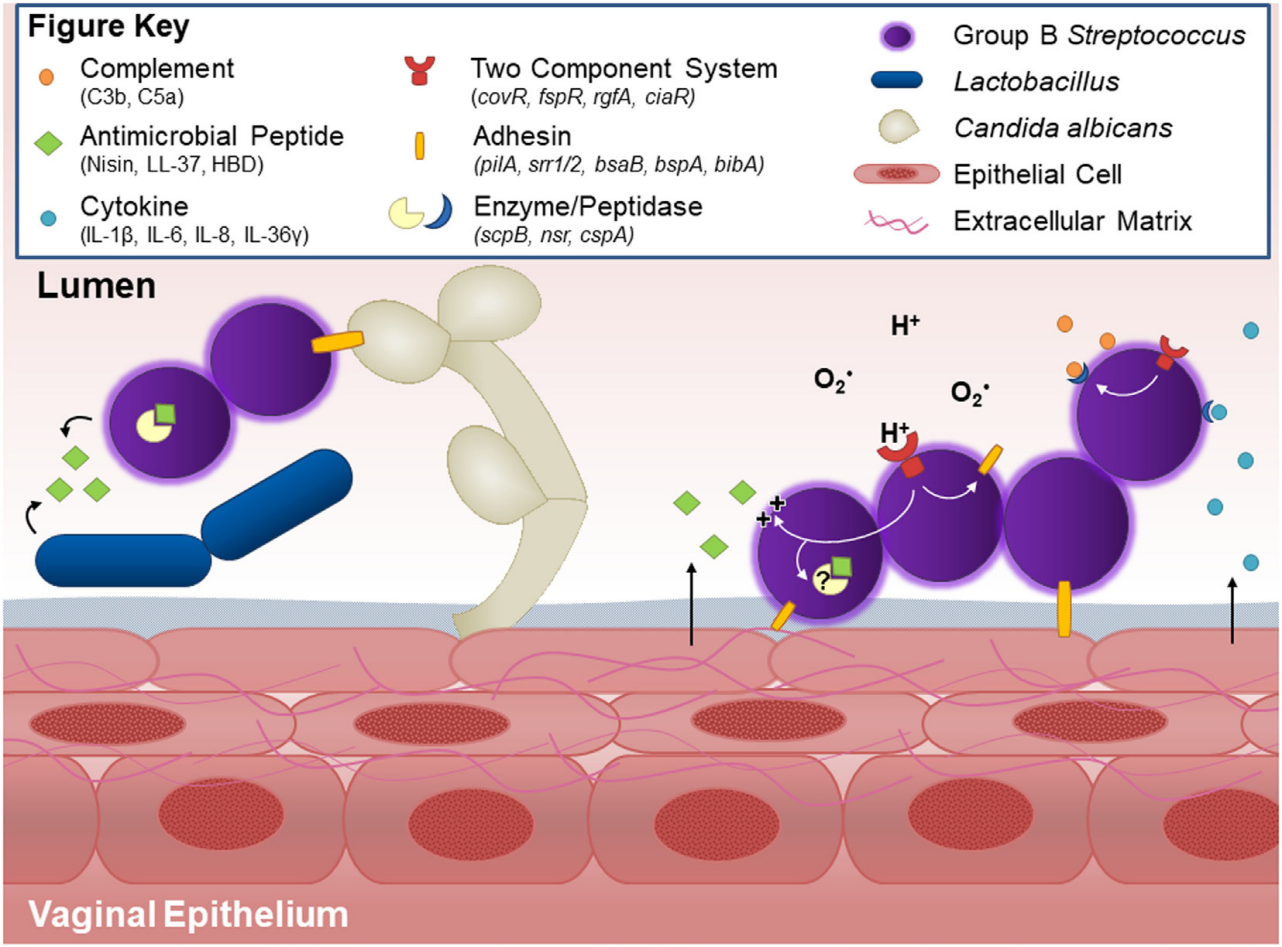
Host and bacterial factors contributing to group B *Streptococcus* (GBS) vaginal colonization. Within the vaginal tract, GBS interacts with other vaginal flora. *Lactobacillus* and GBS both possess antagonistic activity against each other, likely through production of antimicrobial peptides and niche competition. GBS produces countermeasures such as the protease NSR, which degrades the lantibiotic Nisin produced by *Lactococcus*. Other flora, such as *C. albicans*, facilitate GBS vaginal colonization in part through the GBS adhesion BspA. GBS binds to host vaginal epithelial cells and extracellular matrix proteins through surface adhesins including pili, Srr-1 and Srr-2, BsaB, BspA, and BibA. Upon interaction with the epithelium, GBS elicits host cytokine responses such as IL-1β, IL-8, CXCL1, and CXCL2, the latter two of which can be degraded by the serine protease CspA. The vaginal epithelium can also generate antimicrobial peptides, such as LL-37 or β-defensins, which GBS deflects with lipoteichoic acid-anchored d-alanine, or possibly degrades through yet unidentified peptidases. GBS further thwarts innate immune mechanisms by blocking capsular deposition of C3b or through degradation of C5a *via* ScpB. GBS uses multiple two-component systems to sense environmental conditions and regulate virulence and survival factors *via* response regulators such as CovR and CiaR.

The transient nature of GBS vaginal colonization likely reflects a combination of GBS determinants, antagonism by commensal flora, host immune responses, and changes in pregnancy status, vaginal pH and estrous cycle, among other factors which are summarized in Figure 1. One of the most critical steps in successful GBS colonization of the mucosal surface is adherence to luminal epithelial cells and/or surface host proteins. Increased GBS adherence to vaginal epithelial cells and extracellular matrix (ECM) proteins has been observed *in vitro* as pH shifts from acidic to neutral, suggesting a propensity for tissue attachment at vaginal pH (43). Several specific GBS surface-expressed determinants have been shown to contribute to vaginal and cervical epithelial cell adherence, including surface serine-rich repeat (Srr) proteins, Srr-1 and Srr-2 (44, 45), alpha-like proteins (46), the pilus protein PilA of the GBS pilus island (PI)-2a (44), bacterial surface adhesins BsaB (47), BspA (48), and BibA (49). In addition, GBS pili, and other surface proteins, promote adherence to ECM constituents such as collagen (50), fibrinogen (51–53), fibronectin (52, 54–56), and laminin (57, 58), all of which have been identified in multiple vaginal proteome studies (59). Of note, GBS possesses metallopeptidases capable of cleaving all four of these ECM proteins (60), which may aid in local tissue contact and invasion, or niche establishment. GBS constituents apart from surface adherence proteins can influence cervicovaginal adherence or vaginal persistence, including particular capsular serotypes (61, 62), expression of β-hemolysin/cytolysin (β-H/C) toxin and carotenoid pigment (63, 64), and MntH, an H+-dependent manganese transporter (65).

## Biofilm Formation and GBS Vaginal Colonization

Group B *Streptococcus* biofilm development appears to support vaginal tract colonization by affording protection from harsh environmental factors and host defenses (66). Acidic conditions characteristic of the vaginal tract may promote GBS biofilms (67, 68), although some studies have yielded contradictory results (69). Under acidic conditions, some hypervirulent ST-17 strains show heightened biofilm production compared with other sequences types (67), whereas in neutral conditions, neonatal ST-17 and ST-19 strains formed weaker biofilms vs. colonizing isolates (42). In addition, GBS surface proteins PI-2a and FbsC have been implicated in the formation of biofilms *in vitro* (66, 70, 71). GBS vaginal biofilms using *in vivo* models, or in human clinical observations, have not been demonstrated and future development of such models would increase our understanding of GBS colonization at the mucosal surface.

## Influence of Vaginal Microbiota on GBS Colonization

In contemporary schema, the healthy human vaginal microbiome has been clustered into five different communities. Four communities are dominated by *Lactobacillus* species that are believed to lower the environmental pH through lactic acid production, helping protect the host from various microbial pathogens (72). During pregnancy, there is a reduction in species diversity within the vaginal microbiota, with a dominance of *Lactobacillus* species and the orders *Lactobacillales*, followed by *Clostridiales, Bacteroidales*, and *Actinomycetales*, which drive a further lowering vaginal pH to protect both mother and fetus from infection (73, 74). Whether or not GBS should be considered a native component of the vaginal microbiota is still debated. A number of recent reports have described a relative reduction of vaginal *Lactobacillus* populations in GBS-positive women (75–77), although other studies have failed to establish such changes (37, 78). Furthermore, an absence of *Lactobacillus* within the gut has been established as a risk factor for GBS vaginal colonization (31). Interestingly, an inverse relationship between *Lactobacillus* and GBS has also been observed in cows with subclinical mastitis (79). Certain *Lactobacillus* strains have the capacity to inhibit GBS adherence to vaginal epithelial cells (80, 81), and antimicrobial activity of *Lactobacillus* against GBS has been documented *in vitro* (82) and reduction of colonization seen *in vivo* (83, 84). Although the full complexity of the vaginal microbiome is only now being characterized, preliminary *in vitro* studies have begun to probe GBS communication and cooperation with other microbes in this host microenvironment. In pregnant women, GBS is frequently co-isolated with *C. albicans* (28, 76, 85), whereas co-isolation with other pathogens such as *Chlamydia trachomatis, Ureaplasma urealyticum, Trichomonas vaginalis*, and *Mycoplasma hominis* has not been observed (28). In a recent study in non-pregnant women, GBS colonization was positively correlated with vaginal *Prevotella bivia*, and increased rates of GBS colonization were observed in the non-*Lactobacillus* dominated vaginal community state type IV-A (86). GBS binds directly to *C. albicans*, in part through interactions facilitated by the surface-anchored BspA protein, which also assists in epithelial cell adherence (48). GBS also utilizes products derived from vaginal microbes, such as exogenous 1,4-dihydroxy-2-naphthoic acid, to stimulate its own respiratory metabolism (87). Moreover, *in vitro* studies suggest GBS may exchange quorum sensing molecules with other *Streptococcus* species to reciprocally influence each other’s gene expression (88). The presence of GBS may also affect the virulence properties of other reproductive tract pathogens. For example, GBS culture supernatants increase production of toxic shock syndrome toxin 1 in *Staphylococcus aureus* (89), and these two organisms are frequently co-isolated from vaginal swabs (90, 91) as well as the infant nasopharynx (92). GBS possesses several resistance mechanisms for competing with the dominant *Lactobacillus* spp. and other native flora. These include the manganese transporter MntH that supports GBS growth during lactic acid exposure (65). GBS is inherently resistant to the antimicrobial activity of nisin, a lantibiotic produced by *Lactococcus lactis*, through the action of SaNSR, a nisin-degrading enzyme that cleaves off the terminal six amino acids of the peptide to dramatically reduce (100-fold) its bactericidal activity (93, 94). GBS can also inhibit the growth of groups A, B, C, and G streptococci, *Gardnerella vaginalis*, lactobacilli, and diphtheroids under *in vitro* coculture conditions (95). Interestingly, GBS was never isolated with other β-hemolytic streptococci in a clinical study of vaginal swabs (90) or infant nasopharyngeal swabs (92). However, more studies are required to fully elucidate the molecular mechanisms governing GBS persistence and competition among the normal vaginal microbiota.

## Regulatory Systems Influencing GBS Pathogenicity

Group B *Streptococcus* has several genetically encoded regulatory systems in place that impact the transition from a commensal niche (e.g., vaginal or gastrointestinal tract) to invasive niches (e.g., blood, lungs, or brain). Like many bacterial pathogens, GBS respond to changes in environmental stimuli using two-component systems (TCS), which typically consist of a membrane-associated histidine kinase, with a sensor or input domain and an intracellular kinase domain, and a cytoplasmic transcriptional response regulator (96, 97). Sequence analyses reveal that GBS strains typically have 17–21 TCS (98–100), a curious abundance compared with the related important human streptococcal pathogens *S. pneumoniae* (~14 TCS) and *S. pyogenes* (~13 TCS), suggesting that GBS may have a more nuanced capacity to sense and respond to various environmental conditions within the host (98). GBS TCS have important roles in controlling virulence, adherence, resistance to host defenses, and bacterial metabolism. The most well-characterized GBS TCS to date is the sensor histidine kinase CovS (Cov = control of virulence) coupled to response regulator CovR, which coordinately regulate up to 27% of the entire genome (101). CovR/S has largely inhibitory effects on virulence gene expression, including downregulation of fibrinogen-binding proteins A, B, and C (FbsA, FbsB, and FbsC), multiple non-pilus adherence factors, genes involved in iron uptake, and the *cyl* operon implicated in production of the β-H/C toxin and antioxidant carotenoid pigment (43, 66, 102–104). TCS RgfA/C controls expression of the GBS C5a peptidase, which inactivates a critical host complement-derived chemokine, as well as expression of several surface proteins including fibrinogen-binding proteins FbsA and FbsB (99, 105, 106). The recently characterized TCS HssRS senses and regulates heme utilization and metabolism critical for colonization of blood-rich organs (107). TCS CiaR/H promotes GBS oxidant resistance and intracellular trafficking, and by regulating several putative peptidases may enhance GBS resistance to endogenous host antimicrobial peptides (AMPs) (108, 109). Likewise, TCS LiaR/S responds to host AMPs acting on cell wall integrity to modulate cell wall synthesis (110), and TCS DltR/S maintains levels of d-alanine in GBS cell wall lipoteichoic acid (LTA) to increase AMP resistance (111). By coordinately regulating virulence factors, stress response, and AMP sensitivity, GBS mutants deficient in several of the above TCS (with the exception of RgfA/C) have been shown to have attenuated virulence *in vivo*. Conversely, mutation in one TCS BgrR/S that controls expression of the β-antigen (*bac* gene) is associated with increased GBS virulence in a mouse sepsis model (112). With respect to GBS vaginal colonization, only the CovR/S system has been proven to regulate vaginal epithelial cell attachment and promote vaginal persistence *in vivo* (43, 63, 99). Recently identified TCS FspS/R regulates fructose metabolism and plays a role in GBS vaginal colonization (99), but its effect on vaginal epithelial cell attachment *per se* has not been studied. Likewise, TCS NsrR/K senses and regulates resistance genes involved in lantibiotic resistance, which theoretically could enable GBS to better compete against lantibiotic-producing mucosal flora (113), although this has not yet been confirmed *in vivo*.

## GBS Interaction with Host Innate Immune Receptors

Immediate host recognition of invading bacterial pathogens includes complement deposition and engagement of receptors for pathogen-associated molecular patterns (PAMPs), including toll-like receptors (TLRs) (114). GBS has a number of factors to counteract the opsonizing effects of complement, including its surface polysaccharide capsule, which prevents C3b deposition (115, 116), and lessens host production of C5a (117); C5a levels are further degraded by the specific GBS peptidase ScpB (118, 119). GBS also thwarts the complement system through a secreted complement interfering protein (CIP) (120), surface protein BibA (49), and binding of inhibitory complement factor H to the surface-expressed β-protein (Bac) (121) and streptococcal histidine triad (122). TLRs implicated in GBS recognition including TLR2 and TLR6 which engage cell wall LTA and lipoproteins (123, 124), and processes of intracellular sensing by endosomal TLR7 (125) and the murine endosomal TLR13 (124, 126). TLR signaling activates adaptor protein MyD88 and GBS-induced NF-κB translocation, and phagocyte generation of reactive oxygen species (though not phagocytosis itself) is significantly impaired in MyD88−/− mice (127). In macrophages, MyD88 and fellow TLR adaptor UNC-93 signaling elicit the production multiple cytokines in response to GBS (128). Compared with WT mice, TLR2- and MyD88-deficient animals were less able to control systemic GBS infection at lower doses, but conversely, were protected from cytokine storm induced by lethal GBS challenge, highlighting the importance of MyD88 in the magnitude to host cytokine response to the pathogen (129). In contrast to other pyogenic bacterial pathogens such as *S. aureus*, intracellular NOD2 receptor signaling was not critical in host defense against GBS (130, 131).

## GBS Sialic Acid Molecular Mimicry and Siglecs

Another unique mechanism by which GBS evades innate immunity is through molecular mimicry of a critical host glycan. All GBS capsular polysaccharide serotypes present a prominent α2,3-linked terminal sialic acid in their repeat units, which is precisely identical to a common host cell epitope present on glycolipids and glycoproteins decorating the surface of all mammalian cells (132). A family of inhibitory leukocyte receptors, the sialic acid-binding immunoglobulin-like lectins (Siglecs), plays an important role in immune homeostasis by recognizing sialic acid as a “self” epitope, and GBS molecular mimicry allows inhibitory Siglec engagement to downregulate macrophage and neutrophil responses including phagocytosis, oxidative burst, cytokine release and bacterial killing (133–138). Interestingly, the surface-anchored β-protein present in some GBS strains can engage inhibitory Siglec-5 on human macrophages in a sialic-acid-independent manner but still elicit promote inhibitory signaling to suppress innate immune responsiveness (135). Transgenic mice expressing a soluble form of human Siglec-9, that competitively inhibits GBS engagement of the inhibitory Siglec-9 to downregulate neutrophil function, demonstrated improved survival, suggesting a potential novel therapeutic avenue (139).

## Patterns of GBS Cytokine Stimulation

Group B *Streptococcus* cytokine stimulation has been examined in many human immune, epithelial, and endothelial cells that comprise host barriers and defenses including dendritic cells (140), monocytes (141), lung epithelial cells (142), urinary bladder epithelial cells (143), vaginal and cervical epithelial cells (63), brain microvascular endothelial cells (144), astrocytes (145), and coronary artery endothelial cells (146). Intravenous or intraperitoneal GBS infection in mouse models induced robust production of TNF-α, IL-1β, and IL-6 (147, 148); and these cytokines as well as IFN-γ, IL-8, and IL-10 were activated upon intraperitoneal challenge of humanized mice (149). Although important for control of GBS in sublethal challenge doses, TNF-α may be detrimental to the host during GBS septic shock as anti-TNF-α antibody treatment reduced mortality upon high-dose GBS challenge (129, 150). IL-1β contributes to CXCL1 and CXCL2 chemokine signaling resulting in neutrophil recruitment to GBS-infected tissues (151, 152), and neutrophils themselves are a key producer of IL-1β in an amplification loop for innate immunity and inflammation (153). IL-1 receptor signaling contributes to host clearance of GBS, even at sublethal doses (152). IFN-γ, as well as IFN-γ inducers IL-12 and IL-18, protects the host during GBS infection (154–156). Recent studies have shown that blocking IL-10, or IL-10 deficiency, promotes resistance to GBS sepsis in part by restoring neutrophil recruitment to sites of infection (157, 158). The role of particular GBS bacterial components in modulating cytokine responses has been a subject of recent studies. The GBS protease CspA cleaves multiple CXC chemokines, but not CC chemokines (159). GBS single-stranded RNA facilitates macrophage recognition of GBS and subsequent cytokine responses *via* MyD88 (128). Furthermore, GBS RNA and β-H/C induce IL-1β in macrophages through activation of the NLRP3 inflammasome (153, 160). β-H/C is also responsible for increased anti-inflammatory IL-10 production in macrophages, concurrent with decreased IL-12 and NOS2 expression (161). GBS GAPDH has also been linked to host immune modulation through induction of IL-10 (162). Neonatal mononuclear cells produce significantly less of the critical IFN-γ inducing cytokines IL-12 and IL-18 (163), which may be involved in protecting the host as demonstrated by increased survival and reduced GBS load with recombinant IFN-γ treatment in a neonatal sepsis model (154). Furthermore, presence of serum IFN-γ at 20 weeks gestation was more common in women clearing GBS by 37 weeks gestation compared with women who were still colonized (164). Recently, CD4+ T cells were recognized as a source of IFN-γ during GBS-induced sepsis (165).

## Vaginal Immunity to GBS Infection

Studies examining potential host immune responses resulting from asymptomatic GBS vaginal colonization, or from other tissues of the female reproductive tract, are few in number. One study comparing phagocyte engulfment of GBS in colonized and non-colonized pregnant women observed that monocytes from colonized women engulfed significantly more GBS but released more superoxide extracellularly suggesting impaired or insufficient immune function may contribute to GBS vaginal persistence (166). Another work demonstrated that vaginally colonized women possessed elevated levels of IgG and IgA antibodies to GBS in cervical secretions compared with non-colonized women (167). In addition, increased levels of maternal serum IL-1β have been associated with increased risk of GBS neonatal infection and early term birth (168). Vaginal fluid collected from GBS-positive pregnant women contains higher concentrations of MMP-8 and neutrophil gelatinase-associated lipocalin compared with GBS-negative samples (169). Rectal inoculation of GBS in mice stimulated vaginal secretion of IgA (170).

Until recently, GBS induction of host responses of the vaginal epithelium had not been examined. *In vitro* analyses of vaginal and cervical epithelial cells demonstrate robust induction of inflammation including IL-1β production and epithelial disruption by matrix metalloproteinases (MMP) and VEGF, and neutrophil recruitment through IL-8, CXCL1, and CXCL2, as well as inflammatory mediators such as IL-6 and IL-36γ (62, 63). In murine models of vaginal colonization, GBS was found to stimulate IL-1β, IL-6, IL-17, CXCL1, TNF-α, and GM-CSF (62–64, 84). In addition, IL-17+ neutrophils and T cells, and mast cells have been implicated in GBS clearance from the vaginal tract (62, 171).

## Placental Immune Responses to GBS Infection

Since GBS is capable of crossing placental barriers *in utero*, the final physical barrier to the developing fetus, the host responses to GBS within these tissues is actively investigated. Ascension of GBS from the vaginal tract has been observed in both non-pregnant (62) and pregnant mice (172) suggesting this is the main route by which GBS compromises placental tissues. GBS stimulates HBD-2, IL-1β, IL-8, IL-10, and TNF-α in human extraplacental or chorioamniotic membranes *ex vivo* (103, 173–175). In a rhesus monkey GBS infection model, increased amniotic fluid TNF-α, IL-1β, and IL-6 occurred before uterine contractility or any clinical signs of infection, suggesting a direct role for infection in triggering preterm labor (176). Additional non-human primate GBS chorioamionitis studies demonstrated increased amniotic fluid TNF-α, IL-8, IL-1β, IL-6, and fetal IL-8 along with fetal lung injury (177), combined with reduced cytokeratin and other cytoskeletal genes which may compromise fetal membrane integrity (178). In an *in utero* infection model, GBS β-H/C was implicated in GBS-mediated fetal injury through both NLRP3 inflammasome-dependent and -independent pathways (179). In a non-human primate model, a hyperhemolytic GBS mutant induced inflammatory cytokines IL-6 and IL-8 in the amniotic fluid compared with non-hemolytic and uninfected controls (180). In a rat model of GBS-induced chorioamnionitis, IL-1β increased in both placental tissues and fetal blood followed by neutrophil invasion into the placenta (181). Up to one-fourth of invasive GBS infection during pregnancy end in stillbirth or abortion (182). Furthermore, recent work suggests that not only can GBS cause placental dysfunction but also maternal inflammation may affect offspring brain development and neurobehavioral traits particularly in male offspring (181, 183, 184).

## GBS Disease in Pregnancy and *In Utero* Complications

While notorious for its preeminent role in neonatal infections, GBS also causes various maternal infections, with pregnant women displaying an increased incidence of invasive GBS disease, both during gestation (0.04 cases per 1,000 woman-years) and postpartum (0.49 cases per 1,000 woman-years), compared with non-pregnant women (0.02 cases per 1,000 woman-years) (182). GBS carriage is increased in women presenting with vaginitis (185) and in some cases, GBS may even be the etiologic agent of the clinical syndrome (186). GBS bacteremia without focus is also exceptionally prevalent during pregnancy and the immediate postpartum period, accounting for 75% of cases in adults. GBS vaginal colonization, particularly heavy colonization, at the time of delivery has been associated with preterm birth and premature rupture of membranes (PROM) in several individual study populations (9, 187–190), but not in one systematic review (191). GBS ascension of the reproductive tract during pregnancy may result in intra-amniotic infection, chorioamnionitis, or stillbirth (13, 192–195), and GBS burden is increased in placentas from individuals with preterm birth and severe chorioamnionitis (196). Recently, a number of research groups have developed animal models of GBS ascending *in utero* infection to study disease mechanisms. The GBS β-H/C toxin was identified as a key virulence factor contributing to *in utero* infection in several such models. In ascending GBS infection during pregnancy in mice, wild-type GBS instigated more preterm birth and fetal demise, along with increased placental inflammation and fetal bacterial burdens, compared with isogenic β-H/C-deficient mutants (172). Upon direct intrauterine instillation of bacteria, β-H/C was implicated as a driver of GBS-mediated fetal injury in mice (179). Furthermore, in a non-human primate model, hyperhemolytic GBS initiated preterm birth more readily that non-hemolytic GBS controls (180). GBS hyaluronidase was another virulence determinant shown to contribute to bacterial ascension into the uterus, fetal demise, and preterm birth, and further acts to suppress uterine inflammatory responses when live GBS is recovered from uterine horns (197). These animal studies correlate well with human clinical observations regarding the GBS virulence mechanisms at play. Primary human amniotic epithelial cells and intact chorioamniotic membranes are more readily invaded or traversed by a hyperhemolytic Δ*covR* mutant of GBS, compared with wild-type or β-H/C-deficient strains (103), and increased GBS hyaluronidase activity is observed in GBS clinical isolates from preterm birth cases compared with invasive neonatal or colonizing vaginal isolates (197).

## GBS Urinary Tract Infection, Mastitis, and Postpartum Maternal Disease

Group B *Streptococcus* causes approximately 160,000 cases of urinary tract infection (UTI) annually in the U.S. (198). In up to 3.5% of pregnancies, GBS is the etiologic agent of UTI or asymptomatic bacteriuria (199, 200). Left untreated, bacteriuria may advance to acute pyelonephritis, with GBS the leading Gram-positive pathogen, representing 10% of cases in pregnancy (201). GBS bacteriuria during pregnancy is associated with increased risk of intrapartum fever, chorioamnionitis, preterm delivery, and PROM (202), and recently, was found to indicate intrapartum vaginal colonization independent of rectovaginal swab screening (203). Currently, the CDC recommends IAP treatment to all mothers with positive GBS urine cultures during pregnancy (2). The epidemiology, host response, and bacterial virulence factors influencing GBS UTI have been reviewed recently (198). Of note, multiparity is a risk factor for UTI during pregnancy (204), and this multiparous correlation has been demonstrated in GBS UTI model in aged mice (205). A contribution of β-H/C to UTI has been observed in some studies but not others (206, 207), suggesting variables such the GBS strains or murine infection model employed can influence the experimental outcome. In the postpartum period, GBS can cause symptomatic or asymptomatic mastitis in mothers and is proposed as a possible infection route for late-onset neonatal disease (192). GBS may be present in the breastmilk of 3–10% of lactating women, and in up to 20% of women with mastitis (208, 209) Interestingly, mothers who are positive for Lewis antigen system, a blood group which influences the types of human milk oligosaccharides within an individual, are less likely to be vaginally colonized by GBS and to have a colonized infant (210). Whether breastmilk is a source of GBS infant transmission, or infant protection from GBS, is still controversial. Evidence exists for both associations; GBS LOD occurs in some infants from high bacterial loads in breastmilk, yet human milk oligosaccharides are antibacterial against GBS, highlighting the need for further studies to develop recommendations for women with GBS-positive breastmilk (211, 212). Severe maternal infections have also been reported including bacteremia, endometritis, sepsis, and meningitis (192, 213).

## Rational and Efficacy for GBS IAP

By the mid-1990s, the U.S. Centers for Disease Control and Prevention issued the recommendation for intrapartum (intravenous) antibiotic prophylaxis (IAP) to GBS-positive mothers, and in 2002, further recommended universal screening of pregnant women for vaginorectal colonization in weeks 35–37 of gestation (2). Oral antibiotics are not recommended, as no reduction in maternal colonization or neonatal transmission has been observed (214, 215). Current recommendations for GBS neonatal disease prevention consists of universal maternal screening for GBS in the 35–37th week of gestation, with IAP given to GBS-positive mothers during labor (2). Unfortunately, even with screening women just before full term is still not a completely accurate depiction of colonization status at delivery. In one study, over 20% of 37th week GBS-positive women were GBS-negative at the time of delivery (216). In addition, hospital compliance with CDC guidelines confounds the efficacy of IAP with as little as 65% of GBS carriers receiving IAP (217). When given correctly, IAP reduces GBS vaginal colonization to 47% after 2 h of administration, and 12% after 4 h of administration (218). Four hours of IAP with a beta-lactam has been shown to be highly effective in preventing early-onset disease (219) yet there is evidence to support treating for longer than 4 h when possible (220). Another benefit of IAP is that neonates are less likely to be colonized with GBS at birth, although maternal transmission may occur in the months following (221, 222). Furthermore, the maternal vaginal flora, including GBS, does not appear to develop selective antibiotic resistance after IAP administration (223). IAP has led to a dramatic reduction in GBS EOD to approximately 1,000 cases in the U.S. annually. Nevertheless, GBS remains the leading cause of early-onset neonatal sepsis in term infants, and late-onset occurrence remains unaffected, and may be on the rise (2, 224–226). The CDC Active Bacterial Core surveillance program currently estimates a total of 28,150 cases of GBS infection including neonatal and adult populations resulting in 1,865 deaths annually in the U.S. (227).

## Potential Unintended Consequences of IAP

Whether or not IAP alters infection rates of other pathogens or increases GBS antibiotic resistance remains unclear. Some studies have alerted to the possibility of negative IAP effects including increased infections with Gram-negative bacteria such as ampicillin-resistant *E. coli* (228, 229), whereas others have not (230). A recent epidemiological study found an increase in GBS LOD from 1997–2001 to 2002–2010, but it is not known whether this is due to a shift in GBS pathogenicity, or due to an increase in survival of preterm infants, or delay in disease onset from IAP (226). As determined by oral swab, immediate vertical transmission of lactobacilli within hours of birth is reduced in neonates exposed to IAP (231). Several studies have recorded a reduction of fecal *Bifidobacteria* counts in IAP-exposed neonates at 1 week of age, but this difference may be nullified by 1 month of age (232, 233). Levels of gut lactobacilli are not altered by IAP exposure; however, and exclusively breastfed infants have higher *Lactobacillus* counts in the first month of life (233). A combination of IAP and emergency cesarean section may have even more pronounced effects on the infant microbiome (234). In one study, which adjusted for IAP use, infants born to GBS-positive mothers had increased abundance of *Clostridiaceae, Ruminococcaceae*, and *Enterococcaceae* at 6 months of age, suggesting that GBS exposure in and of itself may alter gut composition in early life (235). Furthermore, both mother and infant are more susceptible to fungal infections postpartum when IAP is administered (236), and maternal vaginal flora is altered with exposure to antibiotics (78, 237). Apart from altered infant microbiota, IAP exposure may also predispose neonates to various health and developmental disorders. Children born to women receiving antibiotics for spontaneous preterm labor displayed increased functional impairment as well as an increased risk of cerebral palsy (238). Additional studies have examined antibiotic exposure later in infancy, where it can be speculated that at least some of the health impacts are similar. Infants exposed to prenatal antibiotics or receiving antibiotics within the first 6 months of life display increased risk of childhood obesity, or increased body mass by 2 years of age, respectively (239, 240). Maternal GBS colonization and IAP have also been associated with increased infant aortic intima-media thickness, an early marker for risk of cardiovascular disease (241). Risk of developing childhood asthma is increased with early exposure to antibiotics including administration during infancy, or *in utero* exposure from maternal UTI treatment (16, 242). A reduction in intestinal *Bifidobacteria* has been reported in children with atopic dermatitis (243), similar to the effect seen with IAP in the first 6 months of life; however, no direct correlation has been made between allergy development and maternal IAP or GBS vaginal colonization to date (244). The heightened incidence of allergies and autoimmune diseases in modern Western cultures, particularly with early childhood onset, merits further clarification of long-term impacts of IAP. In addition, adverse maternal and neonatal events resulting from IAP has been recently systematically reviewed (245). The current considerations for IAP to prevent neonatal GBS disease are summarized in Table 1 and highlight the need for more targeted or narrow spectrum prophylaxis.

**Table 1:** Benefits and considerations for maternal intrapartum antibiotic prophylaxis (IAP).

		Reference
**Benefits of IAP**		
Maternal	Twofold to eightfold reduction in vaginal colonization at delivery	(218)
	No increase in antibiotic resistance of vaginal flora	(223)
Neonatal	Drastic reduction (>80%) in early-onset disease	(2, 219, 246, 247)
	Reduction in colonization at birth	(221, 222)
**Considerations for IAP**		
Maternal	Increased risk of fungal infection	(236)
	Altered vaginal flora	(78, 237)
Neonatal	Increased risk of fungal infection	(236)
	Potential increase in Gram-negative infections	(228–230)
	Altered transmission of maternal flora	(231)
	Altered gut flora	(232, 233)
	Increased risk of cerebral palsy	(238)
	Increased aortic intima-media thickness	(241)

## Status of GBS Vaccine Development

In hallmark studies, Lancefield demonstrated that GBS elicits antibody production in the host in a strain-specific manner (21). For decades, we have known that humans generate serum antibodies against the GBS capsule, and these antibodies are specific to a particular serotype (248). Now recent work has attempted to identify all major GBS proteins that elicit the production of human serum antibodies (249). Antibodies mounted to GBS influence disease susceptibility in neonatal infection, as infants born to women with higher levels of anti-GBS IgG were at lower risk for early-onset disease than women with low levels of anti-GBS IgG (250). A recent murine model suggests that mucosal immunization can result in vaginal IgG production combined with enhanced GBS vaginal clearance (251). Moreover, both IgG and IgM type antibody responses are present in infants surviving meningitis, suggesting that the neonatal immune response may also participate in protection (252). Work performed by Drs. Carol Baker, Dennis Kasper and colleagues in the mid-1970s examined production of maternal IgG to GBS capsular polysaccharide types laid the foundation for the development of a GBS vaccine (253). The vaccine strategy that has progressed the furthest is a Novartis/GSK trivalent GBS conjugate vaccine (against serotypes Ia, Ib, and III), which has completed Phase II clinical trials in pregnant women (246), with Phase III trials proposed (254). This trivalent conjugate has achieved significantly higher GBS-specific titers (measured out to 90 days) in infants born to vaccinated mothers compared with placebo controls, without impacting antibody responses to diphtheria and pneumococcal vaccination (255). Furthermore, a recent study has demonstrated a striking negative correlation between GBS antibody titers in cord blood and infant colonization at birth through 90 days of life (256). GBS surface proteins have also been proposed as conserved antigens including pilus proteins (257), Srr-1 (258), alpha C protein (259), Sip, and ScpB (260). Analytic models have determined a GBS vaccine has comparable cost-effectiveness to other pediatric vaccines (261), and a combination of a GBS vaccine with ≥70% efficacy, and IAP for unimmunized women, would prevent more GBS-associated disease than the current screening/IAP at a similar cost (262). The history of vaccine development, analysis of current candidates, obstacles especially within low- and middle-income countries, and future development pathways have just recently been extensively reviewed and discussed (263, 264).

## Alternative Targeted Prevention Strategies Against GBS Colonization

Several alternative strategies to prevent or limit maternal GBS colonization, in place of the current IAP or proposed vaccine candidates, have been recently explored. The first type of strategy consists of applying purified or synthetic compounds with specific antimicrobial or inhibitory activity toward GBS. Potential agents suggested thus far come from various natural or synthetic sources and have not been explored beyond preliminary *in vitro* and animal studies. Multiple plant-derived crude extracts and phytochemicals hinder GBS growth in minimum inhibitory concentration assays (265). For example, plant-based lipids from *Aristolochia longa* and *Bryonia dioïca* show inhibitory activity against GBS *in vitro* (266), as does the vaginal microbicide octylglycerol (267). Bacteriostatic synthetic polymers may represent a barrier for selectively blocking GBS adherence to the vaginal mucosa, while allowing normal constituents of the vaginal flora, such as lactobacilli, to persist (268). Recently, a synthetic peptide mimicking human C5a was shown to be directly bacteriocidal toward GBS and displayed therapeutic *in vivo* activity in both peritonitis and vaginal colonization mouse models (269). Although these compounds show preclinical efficacy in controlling GBS in animal models, it has yet to be established if any of them are feasible or cost-effective for human use. As an alternative to antibiotic treatment, intrapartum chlorhexidine vaginal washes have been considered, but resulted in no significant reduction of EOD, but did significantly lower neonatal colonization (270). Another alternative strategy explores the growing trend of probiotics agents to limit pathogen overgrowth while promoting healthy native vaginal flora (271, 272). To date, the most studied probiotic candidates for controlling GBS are within the *Lactobacillus* genus. Multiple studies have documented the inhibitory activity of lactobacilli on GBS growth *in vitro* including *Lactobacillus rhamnosus, Lactobacillus plantarum, Lactobacillus gasseri, Lactobacillus salivarius*, and *Lactobacillus fermentum* (82, 273–277). Pretreatment with *L reuteri*, but not a combination of *L. gasseri* and *L. salivarius*, reduced GBS vaginal colonization in a murine model (82, 83) indicating some probiotic strains may be more efficacious than others. In a recent human clinical trial, daily oral probiotic treatment of two *Lactobacillus* species, *L. rhamnosus* and *L. reuteri*, to GBS-positive women at the 35–37th week screening visit was found to reduce GBS colonization at the time of delivery (278). Other probiotic species such as *Bifidobacterium*, known to be reduced in the neonatal gut after IAP, have antibacterial activity against GBS (232). The efficacy of a mixture of *Lactobacillus acidophilus, Bifidobacterium lactis*, and *Bifidobacterium longum* has been examined in one small pilot study, and while potentially effective, results did not achieve significant due to modest sample size. Nevertheless, an inverse relationship of yogurt consumption and GBS vaginal colonization was observed (279). Finally, an oral probiotic, *S. salivarius*, was found to inhibit GBS adherence and vaginal colonization in a mouse model (280).

## Conclusion and Perspective

Over the last 50 years, GBS has remained a prominent concern for mother and neonatal health. Although universal screening and IAP have reduced the incidence of early-onset GBS sepsis, maternal and infant colonization rates remain unchanged, and LOD and potential GBS-induced preterm birth are not impacted by IAP. Recent discoveries in the molecular and microbial determinants of GBS vaginal colonization and placental disease have given a better understanding of host–microbe interactions within the female reproductive tract. Furthermore, advances in GBS vaccines and human trials, as well as the emergence of novel targeted strategies to control GBS vaginal colonization, point to a new era beyond broad-spectrum antibiotics, and its detrimental consequences, to prevent neonatal GBS pathogenesis.

## Author Contributions

KP designed the review layout and drafted the manuscript and tables. VN obtained funding and edited and reviewed subsequent drafts of the manuscript.

## Conflict of Interest Statement

The authors declare that the research was conducted in the absence of any commercial or financial relationships that could be construed as a potential conflict of interest.

## References

[1] Doran KS Nizet V. Molecular pathogenesis of neonatal group B streptococcal infection: no longer in its infancy. Mol Microbiol (2004) 54(1):23–31. 10.1111/j.1365-2958.2004.04266.x 15458402

[2] Verani JR McGee L Schrag SJ Division of Bacterial Diseases, National Center for Immunization and Respiratory Diseases, Centers for Disease Control and Prevention (CDC) Prevention of perinatal group B streptococcal disease – revised guidelines from CDC, 2010. MMWR Recomm Rep (2010) 59(RR–10):1–36. 21088663

[3] Maisey HC Doran KS Nizet V. Recent advances in understanding the molecular basis of group B *Streptococcus* virulence. Expert Rev Mol Med (2008) 10:e27. 10.1017/S1462399408000811 18803886 PMC2676346

[4] Edmond KM Kortsalioudaki C Scott S Schrag SJ Zaidi AK Cousens S Group B streptococcal disease in infants aged younger than 3 months: systematic review and meta-analysis. Lancet (2012) 379(9815):547–56. 10.1016/S0140-6736(11)61651-6 22226047

[5] Bedford H de Louvois J Halket S Peckham C Hurley R Harvey D. Meningitis in infancy in England and Wales: follow up at age 5 years. BMJ (2001) 323(7312):533–6. 10.1136/bmj.323.7312.533 11546697 PMC48156

[6] Lawn JE Cousens S Zupan J The Lancet Neonatal Survival Steering Team 4 Million neonatal deaths: when? Where? Why? Lancet (2005) 365(9462):891–900. 10.1016/S0140-6736(05)71048-5 15752534

[7] Simonsen KA Anderson-Berry AL Delair SF Davies HD. Early-onset neonatal sepsis. Clin Microbiol Rev (2014) 27(1):21–47. 10.1128/CMR.00031-13 24396135 PMC3910904

[8] Winn HN. Group B *Streptococcus* infection in pregnancy. Clin Perinatol (2007) 34(3):387–92. 10.1016/j.clp.2007.03.012 17765489

[9] Namavar Jahromi B Poorarian S Poorbarfehee S. The prevalence and adverse effects of group B streptococcal colonization during pregnancy. Arch Iran Med (2008) 11(6):654–7. 18976037

[10] Chan GJ Lee AC Baqui AH Tan J Black RE. Prevalence of early-onset neonatal infection among newborns of mothers with bacterial infection or colonization: a systematic review and meta-analysis. BMC Infect Dis (2015) 15:118. 10.1186/s12879-015-0813-3 25886298 PMC4364328

[11] Boyer KM Gotoff SP. Prevention of early-onset neonatal group B streptococcal disease with selective intrapartum chemoprophylaxis. N Engl J Med (1986) 314(26):1665–9. 10.1056/NEJM198606263142603 3520319

[12] Berardi A Rossi C Guidotti I Vellani G Lugli L Bacchi Reggiani ML Factors associated with intrapartum transmission of group B *Streptococcus*. Pediatr Infect Dis J (2014) 33(12):1211–5. 10.1097/INF.0000000000000439 25037035

[13] Schrag S Gorwitz R Fultz-Butts K Schuchat A Prevention of perinatal group B streptococcal disease: a public health perspective. Centers for Disease Control and Prevention. MMWR Recomm Rep (1996) 45(RR–7):1–24. 8637497

[14] Slogrove AL Goetghebuer T Cotton MF Singer J Bettinger JA. Pattern of infectious morbidity in HIV-exposed uninfected infants and children. Front Immunol (2016) 7:164. 10.3389/fimmu.2016.00164 27199989 PMC4858536

[15] Brigtsen AK Jacobsen AF Dedi L Melby KK Fugelseth D Whitelaw A. Maternal colonization with group B *Streptococcus* is associated with an increased rate of infants transferred to the neonatal intensive care unit. Neonatology (2015) 108(3):157–63. 10.1159/000434716 26182960

[16] Wu P Feldman AS Rosas-Salazar C James K Escobar G Gebretsadik T Relative importance and additive effects of maternal and infant risk factors on childhood asthma. PLoS One (2016) 11(3):e0151705. 10.1371/journal.pone.0151705 27002979 PMC4803347

[17] Bisharat N Crook DW Leigh J Harding RM Ward PN Coffey TJ Hyperinvasive neonatal group B *Streptococcus* has arisen from a bovine ancestor. J Clin Microbiol (2004) 42(5):2161–7. 10.1128/JCM.42.5.2161-2167.2004 15131184 PMC404684

[18] Brochet M Couve E Zouine M Vallaeys T Rusniok C Lamy MC Genomic diversity and evolution within the species *Streptococcus agalactiae*. Microbes Infect (2006) 8(5):1227–43. 10.1016/j.micinf.2005.11.010 16529966

[19] Sun J Fang W Ke B He D Liang Y Ning D Inapparent *Streptococcus agalactiae* infection in adult/commercial tilapia. Sci Rep (2016) 6:26319. 10.1038/srep26319 27215811 PMC4877633

[20] Manning SD Springman AC Million AD Milton NR McNamara SE Somsel PA Association of group B *Streptococcus* colonization and bovine exposure: a prospective cross-sectional cohort study. PLoS One (2010) 5(1):e8795. 10.1371/journal.pone.0008795 20098699 PMC2808344

[21] Lancefield RC. A serological differentiation of specific types of bovine hemolytic streptococci (Group B). J Exp Med (1934) 59(4):441–58. 10.1084/jem.59.4.441 19870257 PMC2132330

[22] Berti F Campisi E Toniolo C Morelli L Crotti S Rosini R Structure of the type IX group B *Streptococcus* capsular polysaccharide and its evolutionary relationship with types V and VII. J Biol Chem (2014) 289(34):23437–48. 10.1074/jbc.M114.567974 24990951 PMC4156066

[23] Phares CR Lynfield R Farley MM Mohle-Boetani J Harrison LH Petit S Epidemiology of invasive group B streptococcal disease in the United States, 1999–2005. JAMA (2008) 299(17):2056–65. 10.1001/jama.299.17.2056 18460666

[24] Jones N Bohnsack JF Takahashi S Oliver KA Chan MS Kunst F Multilocus sequence typing system for group B *Streptococcus*. J Clin Microbiol (2003) 41(6):2530–6. 10.1128/JCM.41.6.2530-2536.2003 12791877 PMC156480

[25] Xia FD Mallet A Caliot E Gao C Trieu-Cuot P Dramsi S. Capsular polysaccharide of Group B *Streptococcus* mediates biofilm formation in the presence of human plasma. Microbes Infect (2015) 17(1):71–6. 10.1016/j.micinf.2014.10.007 25448634

[26] Russell NJ Seale AC O’Driscoll M O’Sullivan C Bianchi-Jassir F Gonzalez-Guarin J Maternal colonization with group B *Streptococcus* and serotype distribution worldwide: systematic review and meta-analyses. Clin Infect Dis (2017) 65(Suppl_2):S100–11. 10.1093/cid/cix658 29117327 PMC5848259

[27] Stoll BJ Schuchat A. Maternal carriage of group B streptococci in developing countries. Pediatr Infect Dis J (1998) 17(6):499–503. 10.1097/00006454-199806000-00013 9655542

[28] Regan JA Klebanoff MA Nugent RP. The epidemiology of group B streptococcal colonization in pregnancy. Vaginal infections and prematurity study group. Obstet Gynecol (1991) 77(4):604–10. 2002986

[29] Kwatra G Cunnington MC Merrall E Adrian PV Ip M Klugman KP Prevalence of maternal colonisation with group B *Streptococcus*: a systematic review and meta-analysis. Lancet Infect Dis (2016) 16(9):1076–84. 10.1016/S1473-3099(16)30055-X 27236858

[30] Alp F Findik D Dagi HT Arslan U Pekin AT Yilmaz SA. Screening and genotyping of group B *Streptococcus* in pregnant and non-pregnant women in Turkey. J Infect Dev Ctries (2016) 10(3):222–6. 10.3855/jidc.6190 27031453

[31] Le Doare K Heath PT. An overview of global GBS epidemiology. Vaccine (2013) 31(Suppl 4):D7–12. 10.1016/j.vaccine.2013.01.009 23973349

[32] Manning SD Lewis MA Springman AC Lehotzky E Whittam TS Davies HD. Genotypic diversity and serotype distribution of group B *Streptococcus* isolated from women before and after delivery. Clin Infect Dis (2008) 46(12):1829–37. 10.1086/588296 18462173 PMC9491394

[33] Khan MA Faiz A Ashshi AM. Maternal colonization of group B *Streptococcus*: prevalence, associated factors and antimicrobial resistance. Ann Saudi Med (2015) 35(6):423–7. 10.5144/0256-4947.2015.423 26657224 PMC6074477

[34] Capan-Melser M Mombo Ngoma G Akerey-Diop D Basra A Wurbel H Groger M Evaluation of intermittent preventive treatment of malaria against group B *Streptococcus* colonization in pregnant women: a nested analysis of a randomized controlled clinical trial of sulfadoxine/pyrimethamine versus mefloquine. J Antimicrob Chemother (2015) 70(6):1898–902. 10.1093/jac/dkv041 25722300

[35] Stapleton RD Kahn JM Evans LE Critchlow CW Gardella CM. Risk factors for group B streptococcal genitourinary tract colonization in pregnant women. Obstet Gynecol (2005) 106(6):1246–52. 10.1097/01.AOG.0000187893.52488.4b 16319248

[36] Akoh CC Pressman EK Cooper E Queenan RA Pillittere J O’Brien KO. Prevalence and risk factors for infections in a pregnant adolescent population. J Pediatr Adolesc Gynecol (2017) 30(1):71–5. 10.1016/j.jpag.2016.08.001 27521899

[37] Brzychczy-Wloch M Pabian W Majewska E Zuk MG Kielbik J Gosiewski T Dynamics of colonization with group B streptococci in relation to normal flora in women during subsequent trimesters of pregnancy. New Microbiol (2014) 37(3):307–19. 25180845

[38] Ferrieri P Baker CJ Hillier SL Flores AE. Diversity of surface protein expression in group B streptococcal colonizing & invasive isolates. Indian J Med Res (2004) 119(Suppl):191–6. 15232193

[39] Edwards MS Rench MA Palazzi DL Baker CJ. Group B streptococcal colonization and serotype-specific immunity in healthy elderly persons. Clin Infect Dis (2005) 40(3):352–7. 10.1086/426820 15668856

[40] Kwatra G Adrian PV Shiri T Buchmann EJ Cutland CL Madhi SA. Serotype-specific acquisition and loss of group B *Streptococcus* recto-vaginal colonization in late pregnancy. PLoS One (2014) 9(6):e98778. 10.1371/journal.pone.0098778 24979575 PMC4076185

[41] Teatero S Ferrieri P Martin I Demczuk W McGeer A Fittipaldi N. Serotype distribution, population structure, and antimicrobial resistance of group B *Streptococcus* strains recovered from colonized pregnant women. J Clin Microbiol (2017) 55(2):412–22. 10.1128/JCM.01615-16 27852675 PMC5277510

[42] Parker RE Laut C Gaddy JA Zadoks RN Davies HD Manning SD. Association between genotypic diversity and biofilm production in group B *Streptococcus*. BMC Microbiol (2016) 16:86. 10.1186/s12866-016-0704-9 27206613 PMC4875601

[43] Park SE Jiang S Wessels MR. CsrRS and environmental pH regulate group B *Streptococcus* adherence to human epithelial cells and extracellular matrix. Infect Immun (2012) 80(11):3975–84. 10.1128/IAI.00699-12 22949550 PMC3486057

[44] Sheen TR Jimenez A Wang NY Banerjee A van Sorge NM Doran KS. Serine-rich repeat proteins and pili promote *Streptococcus agalactiae* colonization of the vaginal tract. J Bacteriol (2011) 193(24):6834–42. 10.1128/JB.00094-11 21984789 PMC3232834

[45] Wang NY Patras KA Seo HS Cavaco CK Rosler B Neely MN Group B streptococcal serine-rich repeat proteins promote interaction with fibrinogen and vaginal colonization. J Infect Dis (2014) 210(6):982–91. 10.1093/infdis/jiu151 24620021 PMC4192050

[46] Baron MJ Bolduc GR Goldberg MB Auperin TC Madoff LC. Alpha C protein of group B *Streptococcus* binds host cell surface glycosaminoglycan and enters cells by an actin-dependent mechanism. J Biol Chem (2004) 279(23):24714–23. 10.1074/jbc.M402164200 15044471

[47] Jiang S Wessels MR. BsaB, a novel adherence factor of group B *Streptococcus*. Infect Immun (2014) 82(3):1007–16. 10.1128/IAI.01014-13 24343649 PMC3957996

[48] Rego S Heal TJ Pidwill GR Till M Robson A Lamont RJ Structural and functional analysis of cell wall-anchored polypeptide adhesin BspA in *Streptococcus agalactiae*. J Biol Chem (2016) 291(31):15985–6000. 10.1074/jbc.M116.726562 27311712 PMC4965550

[49] Santi I Scarselli M Mariani M Pezzicoli A Masignani V Taddei A BibA: a novel immunogenic bacterial adhesin contributing to group B *Streptococcus* survival in human blood. Mol Microbiol (2007) 63(3):754–67. 10.1111/j.1365-2958.2006.05555.x 17212592

[50] Banerjee A Kim BJ Carmona EM Cutting AS Gurney MA Carlos C Bacterial Pili exploit integrin machinery to promote immune activation and efficient blood-brain barrier penetration. Nat Commun (2011) 2:462. 10.1038/ncomms1474 21897373 PMC3195231

[51] Schubert A Zakikhany K Schreiner M Frank R Spellerberg B Eikmanns BJ A fibrinogen receptor from group B *Streptococcus* interacts with fibrinogen by repetitive units with novel ligand binding sites. Mol Microbiol (2002) 46(2):557–69. 10.1046/j.1365-2958.2002.03177.x 12406229

[52] Dehbashi S Pourmand MR Mashhadi R. Characterization of Afb, a novel bifunctional protein in *Streptococcus agalactiae*. Iran J Microbiol (2016) 8(1):73–9. 27092228 PMC4833744

[53] Devi AS Ponnuraj K. Cloning, expression, purification and ligand binding studies of novel fibrinogen-binding protein FbsB of *Streptococcus agalactiae*. Protein Expr Purif (2010) 74(2):148–55. 10.1016/j.pep.2010.07.004 20667474

[54] Tamura GS Rubens CE. Group B streptococci adhere to a variant of fibronectin attached to a solid phase. Mol Microbiol (1995) 15(3):581–9. 10.1111/j.1365-2958.1995.tb02271.x 7783628

[55] Hull JR Tamura GS Castner DG. Interactions of the streptococcal C5a peptidase with human fibronectin. Acta Biomater (2008) 4(3):504–13. 10.1016/j.actbio.2008.01.009 18313373 PMC2409115

[56] Cheng Q Stafslien D Purushothaman SS Cleary P. The group B streptococcal C5a peptidase is both a specific protease and an invasin. Infect Immun (2002) 70(5):2408–13. 10.1128/IAI.70.5.2408-2413.2002 11953377 PMC127948

[57] Franken C Haase G Brandt C Weber-Heynemann J Martin S Lammler C Horizontal gene transfer and host specificity of beta-haemolytic streptococci: the role of a putative composite transposon containing scpB and lmb. Mol Microbiol (2001) 41(4):925–35. 10.1046/j.1365-2958.2001.02563.x 11532154

[58] Spellerberg B Rozdzinski E Martin S Weber-Heynemann J Schnitzler N Lutticken R Lmb, a protein with similarities to the LraI adhesin family, mediates attachment of *Streptococcus agalactiae* to human laminin. Infect Immun (1999) 67(2):871–8. 9916102 10.1128/iai.67.2.871-878.1999 PMC96398

[59] Zegels G Van Raemdonck GA Coen EP Tjalma WA Van Ostade XW. Comprehensive proteomic analysis of human cervical-vaginal fluid using colposcopy samples. Proteome Sci (2009) 7:17. 10.1186/1477-5956-7-17 19374746 PMC2678104

[60] Soares GC da Silva BA Dos Santos MH da Costa AF Dos Santos AL Morandi V Metallopeptidases produced by group B *Streptococcus*: influence of proteolytic inhibitors on growth and on interaction with human cell lineages. Int J Mol Med (2008) 22(1):119–25. 10.3892/ijmm.22.1.119 18575784

[61] Bodaszewska-Lubas M Brzychczy-Wloch M Adamski P Gosiewski T Strus M Heczko PB. Adherence of group B streptococci to human rectal and vaginal epithelial cell lines in relation to capsular polysaccharides as well as alpha-like protein genes – pilot study. Pol J Microbiol (2013) 62(1):85–90. 23829083

[62] Patras KA Rosler B Thoman ML Doran KS. Characterization of host immunity during persistent vaginal colonization by group B *Streptococcus*. Mucosal Immunol (2015) 8(6):1339–48. 10.1038/mi.2015.23 25850655 PMC4598252

[63] Patras KA Wang NY Fletcher EM Cavaco CK Jimenez A Garg M Group B *Streptococcus* covR regulation modulates host immune signalling pathways to promote vaginal colonization. Cell Microbiol (2013) 15(7):1154–67. 10.1111/cmi.12105 23298320 PMC3657335

[64] Carey AJ Tan CK Mirza S Irving-Rodgers H Webb RI Lam A Infection and cellular defense dynamics in a novel 17beta-estradiol murine model of chronic human group B *Streptococcus* genital tract colonization reveal a role for hemolysin in persistence and neutrophil accumulation. J Immunol (2014) 192(4):1718–31. 10.4049/jimmunol.1202811 24453257

[65] Shabayek S Bauer R Mauerer S Mizaikoff B Spellerberg B A streptococcal NRAMP homologue is crucial for the survival of *Streptococcus agalactiae* under low pH conditions. Mol Microbiol (2016) 100(4):589–606. 10.1111/mmi.13335 27150893

[66] Rosini R Margarit I. Biofilm formation by *Streptococcus agalactiae*: influence of environmental conditions and implicated virulence factors. Front Cell Infect Microbiol (2015) 5:6. 10.3389/fcimb.2015.00006 25699242 PMC4316791

[67] D’Urzo N Martinelli M Pezzicoli A De Cesare V Pinto V Margarit I Acidic pH strongly enhances in vitro biofilm formation by a subset of hypervirulent ST-17 *Streptococcus agalactiae* strains. Appl Environ Microbiol (2014) 80(7):2176–85. 10.1128/AEM.03627-13 24487536 PMC3993151

[68] Ho YR Li CM Yu CH Lin YJ Wu CM Harn IC The enhancement of biofilm formation in Group B streptococcal isolates at vaginal pH. Med Microbiol Immunol (2013) 202(2):105–15. 10.1007/s00430-012-0255-0 22797522

[69] Borges S Silva J Teixeira P. Survival and biofilm formation by Group B streptococci in simulated vaginal fluid at different pHs. Antonie Van Leeuwenhoek (2012) 101(3):677–82. 10.1007/s10482-011-9666-y 22038130

[70] Rinaudo CD Rosini R Galeotti CL Berti F Necchi F Reguzzi V Specific involvement of pilus type 2a in biofilm formation in group B *Streptococcus*. PLoS One (2010) 5(2):e9216. 10.1371/journal.pone.0009216 20169161 PMC2821406

[71] Buscetta M Papasergi S Firon A Pietrocola G Biondo C Mancuso G FbsC, a novel fibrinogen-binding protein, promotes S*treptococcus agalactiae*-host cell interactions. J Biol Chem (2014) 289(30):21003–15. 10.1074/jbc.M114.553073 24904056 PMC4110306

[72] Ravel J Gajer P Abdo Z Schneider GM Koenig SS McCulle SL Vaginal microbiome of reproductive-age women. Proc Natl Acad Sci U S A (2011) 108(Suppl 1):4680–7. 10.1073/pnas.1002611107 20534435 PMC3063603

[73] Nuriel-Ohayon M Neuman H Koren O. Microbial changes during pregnancy, birth, and infancy. Front Microbiol (2016) 7:1031. 10.3389/fmicb.2016.01031 27471494 PMC4943946

[74] Aagaard K Riehle K Ma J Segata N Mistretta TA Coarfa C A metagenomic approach to characterization of the vaginal microbiome signature in pregnancy. PLoS One (2012) 7(6):e36466. 10.1371/journal.pone.0036466 22719832 PMC3374618

[75] Kubota T Nojima M Itoh S. Vaginal bacterial flora of pregnant women colonized with group B *Streptococcus*. J Infect Chemother (2002) 8(4):326–30. 10.1007/s10156-002-0190-x 12525892

[76] Altoparlak U Kadanali A Kadanali S Genital flora in pregnancy and its association with group B streptococcal colonization. Int J Gynaecol Obstet (2004) 87(3):245–6. 10.1016/j.ijgo.2004.08.006 15548398

[77] Ronnqvist PD Forsgren-Brusk UB Grahn-Hakansson EE. Lactobacilli in the female genital tract in relation to other genital microbes and vaginal pH. Acta Obstet Gynecol Scand (2006) 85(6):726–35. 10.1080/00016340600578357 16752267

[78] Rick AM Aguilar A Cortes R Gordillo R Melgar M Samayoa-Reyes G Group B streptococci colonization in pregnant Guatemalan women: prevalence, risk factors, and vaginal microbiome. Open Forum Infect Dis (2017) 4(1):ofx020. 10.1093/ofid/ofx020 28480290 PMC5414013

[79] Qiao J Kwok L Zhang J Gao P Zheng Y Guo Z Reduction of *Lactobacillus* in the milks of cows with subclinical mastitis. Benef Microbes (2015) 6(4):485–90. 10.3920/BM2014.0077 25711409

[80] Zarate G Nader-Macias ME. Influence of probiotic vaginal lactobacilli on in vitro adhesion of urogenital pathogens to vaginal epithelial cells. Lett Appl Microbiol (2006) 43(2):174–80. 10.1111/j.1472-765X.2006.01934.x 16869901

[81] Ortiz L Ruiz F Pascual L Barberis L. Effect of two probiotic strains of *Lactobacillus* on in vitro adherence of *Listeria monocytogenes, Streptococcus agalactiae*, and *Staphylococcus aureus* to vaginal epithelial cells. Curr Microbiol (2014) 68(6):679–84. 10.1007/s00284-014-0524-9 24469557

[82] De Gregorio PR Juarez Tomas MS Leccese Terraf MC Nader-Macias ME. In vitro and in vivo effects of beneficial vaginal lactobacilli on pathogens responsible for urogenital tract infections. J Med Microbiol (2014) 63(Pt 5):685–96. 10.1099/jmm.0.069401-0 24523160

[83] De Gregorio PR Juarez Tomas MS Leccese Terraf MC Nader-Macias ME. Preventive effect of *Lactobacillus reuteri* CRL1324 on Group B *Streptococcus* vaginal colonization in an experimental mouse model. J Appl Microbiol (2015) 118(4):1034–47. 10.1111/jam.12739 25786121

[84] De Gregorio PR Juarez Tomas MS Nader-Macias ME. Immunomodulation of *Lactobacillus reuteri* CRL1324 on group B *Streptococcus* vaginal colonization in a murine experimental model. Am J Reprod Immunol (2016) 75(1):23–35. 10.1111/aji.12445 26547516

[85] Bayo M Berlanga M Agut M. Vaginal microbiota in healthy pregnant women and prenatal screening of group B streptococci (GBS). Int Microbiol (2002) 5(2):87–90. 10.1007/s10123-002-0064-1 12180785

[86] Rosen G Randis TM Desai PV Sapra KJ Ma B Gajer P Group B *Streptococcus* and the vaginal microbiota. J Infect Dis (2017) 216(6):744–51. 10.1093/infdis/jix395 28934437 PMC5853324

[87] Franza T Delavenne E Derre-Bobillot A Juillard V Boulay M Demey E A partial metabolic pathway enables group B *Streptococcus* to overcome quinone deficiency in a host bacterial community. Mol Microbiol (2016) 102(1):81–91. 10.1111/mmi.13447 27328751

[88] Cook LC LaSarre B Federle MJ. Interspecies communication among commensal and pathogenic streptococci. MBio (2013) 4(4):e00382–413. 10.1128/mBio.00382-13 23882015 PMC3735184

[89] MacPhee RA Miller WL Gloor GB McCormick JK Hammond JA Burton JP Influence of the vaginal microbiota on toxic shock syndrome toxin 1 production by *Staphylococcus aureus*. Appl Environ Microbiol (2013) 79(6):1835–42. 10.1128/AEM.02908-12 23315732 PMC3592239

[90] Carson HJ Lapoint PG Monif GR. Interrelationships within the bacterial flora of the female genital tract. Infect Dis Obstet Gynecol (1997) 5(4):303–9. 10.1155/S1064744997000525 18476156 PMC2364555

[91] Ghanim N Alchyib O Morrish D Tompkins D Julliard K Visconti E Maternal-neonatal outcome with *Staphylococcus aureus* rectovaginal colonization. J Reprod Med (2011) 56(9–10):421–4. 22010527

[92] Foster-Nyarko E Kwambana B Aderonke O Ceesay F Jarju S Bojang A Associations between nasopharyngeal carriage of group B *Streptococcus* and other respiratory pathogens during early infancy. BMC Microbiol (2016) 16:97. 10.1186/s12866-016-0714-7 27230066 PMC4882866

[93] Khosa S AlKhatib Z Smits SH. NSR from *Streptococcus agalactiae* confers resistance against nisin and is encoded by a conserved nsr operon. Biol Chem (2013) 394(11):1543–9. 10.1515/hsz-2013-0167 23893686

[94] Khosa S Lagedroste M Smits SH Protein defense systems against the lantibiotic nisin: function of the immunity protein NisI and the resistance protein NSR. Front Microbiol (2016) 7:504 10.3389/fmicb.2016.00504 27148193 PMC4828448

[95] Chaisilwattana P Monif GR. In vitro ability of the group B streptococci to inhibit Gram-positive and Gram-variable constituents of the bacterial flora of the female genital tract. Infect Dis Obstet Gynecol (1995) 3(3):91–7. 10.1155/S1064744995000391 18476028 PMC2364431

[96] Beier D Gross R. Regulation of bacterial virulence by two-component systems. Curr Opin Microbiol (2006) 9(2):143–52. 10.1016/j.mib.2006.01.005 16481212

[97] Mascher T Helmann JD Unden G. Stimulus perception in bacterial signal-transducing histidine kinases. Microbiol Mol Biol Rev (2006) 70(4):910–38. 10.1128/MMBR.00020-06 17158704 PMC1698512

[98] Glaser P Rusniok C Buchrieser C Chevalier F Frangeul L Msadek T Genome sequence of *Streptococcus agalactiae*, a pathogen causing invasive neonatal disease. Mol Microbiol (2002) 45(6):1499–513. 10.1046/j.1365-2958.2002.03126.x 12354221

[99] Faralla C Metruccio MM De Chiara M Mu R Patras KA Muzzi A Analysis of two-component systems in group B *Streptococcus* shows that RgfAC and the novel FspSR modulate virulence and bacterial fitness. MBio (2014) 5(3):e870–814. 10.1128/mBio.00870-14 24846378 PMC4030450

[100] Tettelin H Masignani V Cieslewicz MJ Eisen JA Peterson S Wessels MR Complete genome sequence and comparative genomic analysis of an emerging human pathogen, serotype V *Streptococcus agalactiae*. Proc Natl Acad Sci U S A (2002) 99(19):12391–6. 10.1073/pnas.182380799 12200547 PMC129455

[101] Di Palo B Rippa V Santi I Brettoni C Muzzi A Metruccio MM Adaptive response of group B *Streptococcus* to high glucose conditions: new insights on the CovRS regulation network. PLoS One (2013) 8(4):e61294. 10.1371/journal.pone.0061294 23585887 PMC3621830

[102] Lembo A Gurney MA Burnside K Banerjee A de los Reyes M Connelly JE Regulation of CovR expression in group B *Streptococcus* impacts blood-brain barrier penetration. Mol Microbiol (2010) 77(2):431–43. 10.1111/j.1365-2958.2010.07215.x 20497331 PMC2909351

[103] Whidbey C Harrell MI Burnside K Ngo L Becraft AK Iyer LM A hemolytic pigment of group B *Streptococcus* allows bacterial penetration of human placenta. J Exp Med (2013) 210(6):1265–81. 10.1084/jem.20122753 23712433 PMC3674703

[104] Jiang SM Ishmael N Dunning Hotopp J Puliti M Tissi L Kumar N Variation in the group B *Streptococcus* CsrRS regulon and effects on pathogenicity. J Bacteriol (2008) 190(6):1956–65. 10.1128/JB.01677-07 18203834 PMC2258897

[105] Al Safadi R Mereghetti L Salloum M Lartigue MF Virlogeux-Payant I Quentin R Two-component system RgfA/C activates the *fbsB* gene encoding major fibrinogen-binding protein in highly virulent CC17 clone group B *Streptococcus*. PLoS One (2011) 6(2):e14658. 10.1371/journal.pone.0014658 21326613 PMC3033900

[106] Spellerberg B Rozdzinski E Martin S Weber-Heynemann J Lutticken R. rgf encodes a novel two-component signal transduction system of *Streptococcus agalactiae*. Infect Immun (2002) 70(5):2434–40. 10.1128/IAI.70.5.2434-2440.2002 11953380 PMC127907

[107] Joubert L Dagieu JB Fernandez A Derre-Bobillot A Borezee-Durant E Fleurot I Visualization of the role of host heme on the virulence of the heme auxotroph *Streptococcus agalactiae*. Sci Rep (2017) 7:40435. 10.1038/srep40435 28091535 PMC5238366

[108] Quach D van Sorge NM Kristian SA Bryan JD Shelver DW Doran KS. The CiaR response regulator in group B *Streptococcus* promotes intracellular survival and resistance to innate immune defenses. J Bacteriol (2009) 191(7):2023–32. 10.1128/JB.01216-08 19114476 PMC2655536

[109] Mu R Cutting AS Del Rosario Y Villarino N Stewart L Weston TA Identification of CiaR regulated genes that promote group B streptococcal virulence and interaction with brain endothelial cells. PLoS One (2016) 11(4):e0153891. 10.1371/journal.pone.0153891 27100296 PMC4839699

[110] Klinzing DC Ishmael N Dunning Hotopp JC Tettelin H Shields KR Madoff LC The two-component response regulator LiaR regulates cell wall stress responses, pili expression and virulence in group B *Streptococcus*. Microbiology (2013) 159(Pt 7):1521–34. 10.1099/mic.0.064444-0 23704792 PMC3749725

[111] Poyart C Lamy MC Boumaila C Fiedler F Trieu-Cuot P. Regulation of D-alanyl-lipoteichoic acid biosynthesis in *Streptococcus agalactiae* involves a novel two-component regulatory system. J Bacteriol (2001) 183(21):6324–34. 10.1128/JB.183.21.6324-6334.2001 11591677 PMC100127

[112] Rozhdestvenskaya AS Totolian AA Dmitriev AV. Inactivation of DNA-binding response regulator Sak189 abrogates beta-antigen expression and affects virulence of *Streptococcus agalactiae*. PLoS One (2010) 5(4):e10212. 10.1371/journal.pone.0010212 20419089 PMC2856668

[113] Khosa S Hoeppner A Gohlke H Schmitt L Smits SH. Structure of the response regulator NsrR from *Streptococcus agalactiae*, which is involved in lantibiotic resistance. PLoS One (2016) 11(3):e0149903. 10.1371/journal.pone.0149903 26930060 PMC4773095

[114] Kenzel S Henneke P. The innate immune system and its relevance to neonatal sepsis. Curr Opin Infect Dis (2006) 19(3):264–70. 10.1097/01.qco.0000224821.27482.bd 16645488

[115] Marques MB Kasper DL Pangburn MK Wessels MR. Prevention of C3 deposition by capsular polysaccharide is a virulence mechanism of type III group B streptococci. Infect Immun (1992) 60(10):3986–93. 1398910 10.1128/iai.60.10.3986-3993.1992 PMC257427

[116] Edwards MS Kasper DL Jennings HJ Baker CJ Nicholson-Weller A Capsular sialic acid prevents activation of the alternative complement pathway by type III, group B streptococci. J Immunol (1982) 128(3):1278–83. 7035562

[117] Takahashi S Aoyagi Y Adderson EE Okuwaki Y Bohnsack JF. Capsular sialic acid limits C5a production on type III group B streptococci. Infect Immun (1999) 67(4):1866–70. 10085029 10.1128/iai.67.4.1866-1870.1999 PMC96539

[118] Cleary PP Handley J Suvorov AN Podbielski A Ferrieri P. Similarity between the group B and A streptococcal C5a peptidase genes. Infect Immun (1992) 60(10):4239–44. 1398935 10.1128/iai.60.10.4239-4244.1992 PMC257458

[119] Bohnsack JF Mollison KW Buko AM Ashworth JC Hill HR. Group B streptococci inactivate complement component C5a by enzymic cleavage at the C-terminus. Biochem J (1991) 273(Pt 3):635–40. 10.1042/bj2730635 1996961 PMC1149811

[120] Pietrocola G Rindi S Rosini R Buccato S Speziale P Margarit I. The group B *Streptococcus*-secreted protein CIP interacts with C4, preventing C3b deposition via the lectin and classical complement pathways. J Immunol (2016) 196(1):385–94. 10.4049/jimmunol.1501954 26608922 PMC4683360

[121] Areschoug T Stalhammar-Carlemalm M Karlsson I Lindahl G. Streptococcal beta protein has separate binding sites for human factor H and IgA-Fc. J Biol Chem (2002) 277(15):12642–8. 10.1074/jbc.M112072200 11812795

[122] Maruvada R Prasadarao NV Rubens CE. Acquisition of factor H by a novel surface protein on group B *Streptococcus* promotes complement degradation. FASEB J (2009) 23(11):3967–77. 10.1096/fj.09-138149 19608625 PMC2775014

[123] Henneke P Morath S Uematsu S Weichert S Pfitzenmaier M Takeuchi O Role of lipoteichoic acid in the phagocyte response to group B *Streptococcus*. J Immunol (2005) 174(10):6449–55. 10.4049/jimmunol.174.10.6449 15879147

[124] Kolter J Feuerstein R Spoeri E Gharun K Elling R Trieu-Cuot P Streptococci engage TLR13 on myeloid cells in a site-specific fashion. J Immunol (2016) 196(6):2733–41. 10.4049/jimmunol.1501014 26873993

[125] Mancuso G Gambuzza M Midiri A Biondo C Papasergi S Akira S Bacterial recognition by TLR7 in the lysosomes of conventional dendritic cells. Nat Immunol (2009) 10(6):587–94. 10.1038/ni.1733 19430477

[126] Signorino G Mohammadi N Patane F Buscetta M Venza M Venza I Role of toll-like receptor 13 in innate immune recognition of group B streptococci. Infect Immun (2014) 82(12):5013–22. 10.1128/IAI.02282-14 25225249 PMC4249301

[127] Henneke P Takeuchi O Malley R Lien E Ingalls RR Freeman MW Cellular activation, phagocytosis, and bactericidal activity against group B *Streptococcus* involve parallel myeloid differentiation factor 88-dependent and independent signaling pathways. J Immunol (2002) 169(7):3970–7. 10.4049/jimmunol.169.7.3970 12244198

[128] Deshmukh SD Kremer B Freudenberg M Bauer S Golenbock DT Henneke P. Macrophages recognize streptococci through bacterial single-stranded RNA. EMBO Rep (2011) 12(1):71–6. 10.1038/embor.2010.189 21164516 PMC3024127

[129] Mancuso G Midiri A Beninati C Biondo C Galbo R Akira S Dual role of TLR2 and myeloid differentiation factor 88 in a mouse model of invasive group B streptococcal disease. J Immunol (2004) 172(10):6324–9. 10.4049/jimmunol.172.10.6324 15128822

[130] Lemire P Calzas C Segura M The NOD2 receptor does not play a major role in the pathogenesis of Group B *Streptococcus* in mice. Microb Pathog (2013) 65:41–7. 10.1016/j.micpath.2013.09.006 24107312

[131] Lemire P Roy D Fittipaldi N Okura M Takamatsu D Bergman E Implication of TLR- but not of NOD2-signaling pathways in dendritic cell activation by group B *Streptococcus* serotypes III and V. PLoS One (2014) 9(12):e113940. 10.1371/journal.pone.0113940 25436906 PMC4250082

[132] Wessels MR Rubens CE Benedi VJ Kasper DL. Definition of a bacterial virulence factor: sialylation of the group B streptococcal capsule. Proc Natl Acad Sci U S A (1989) 86(22):8983–7. 10.1073/pnas.86.22.8983 2554337 PMC298416

[133] Carlin AF Lewis AL Varki A Nizet V. Group B streptococcal capsular sialic acids interact with siglecs (immunoglobulin-like lectins) on human leukocytes. J Bacteriol (2007) 189(4):1231–7. 10.1128/JB.01155-06 16997964 PMC1797352

[134] Carlin AF Uchiyama S Chang YC Lewis AL Nizet V Varki A. Molecular mimicry of host sialylated glycans allows a bacterial pathogen to engage neutrophil Siglec-9 and dampen the innate immune response. Blood (2009) 113(14):3333–6. 10.1182/blood-2008-11-187302 19196661 PMC2665898

[135] Carlin AF Chang YC Areschoug T Lindahl G Hurtado-Ziola N King CC Group B *Streptococcus* suppression of phagocyte functions by protein-mediated engagement of human Siglec-5. J Exp Med (2009) 206(8):1691–9. 10.1084/jem.20090691 19596804 PMC2722167

[136] Chang YC Olson J Beasley FC Tung C Zhang J Crocker PR Group B *Streptococcus* engages an inhibitory Siglec through sialic acid mimicry to blunt innate immune and inflammatory responses in vivo. PLoS Pathog (2014) 10(1):e1003846. 10.1371/journal.ppat.1003846 24391502 PMC3879367

[137] Ali SR Fong JJ Carlin AF Busch TD Linden R Angata T Siglec-5 and Siglec-14 are polymorphic paired receptors that modulate neutrophil and amnion signaling responses to group B *Streptococcus*. J Exp Med (2014) 211(6):1231–42. 10.1084/jem.20131853 24799499 PMC4042635

[138] Chang YC Nizet V. The interplay between Siglecs and sialylated pathogens. Glycobiology (2014) 24(9):818–25. 10.1093/glycob/cwu067 24996821 PMC4168292

[139] Saito M Yamamoto S Ozaki K Tomioka Y Suyama H Morimatsu M A soluble form of Siglec-9 provides a resistance against group B *Streptococcus* (GBS) infection in transgenic mice. Microb Pathog (2016) 99:106–10. 10.1016/j.micpath.2016.08.014 27544323

[140] Lemire P Houde M Lecours MP Fittipaldi N Segura M Role of capsular polysaccharide in group B *Streptococccus* interactions with dendritic cells. Microbes Infect (2012) 14(12):1064–76. 10.1016/j.micinf.2012.05.015 22683668

[141] De Francesco MA Gargiulo F Negrini R Gelmi M Manca N. Different sequence strains of *Streptococcus agalactiae* elicit various levels of cytokine production. Immunol Invest (2008) 37(8):741–51. 10.1080/08820130802403283 18991093

[142] Mikamo H Johri AK Paoletti LC Madoff LC Onderdonk AB. Adherence to, invasion by, and cytokine production in response to serotype VIII group B Streptococci. Infect Immun (2004) 72(8):4716–22. 10.1128/IAI.72.8.4716-4722.2004 15271933 PMC470694

[143] Ulett GC Webb RI Ulett KB Cui X Benjamin WH Crowley M Group B *Streptococcus* (GBS) urinary tract infection involves binding of GBS to bladder uroepithelium and potent but GBS-specific induction of interleukin 1alpha. J Infect Dis (2010) 201(6):866–70. 10.1086/650696 20132033

[144] Doran KS Liu GY Nizet V. Group B streptococcal beta-hemolysin/cytolysin activates neutrophil signaling pathways in brain endothelium and contributes to development of meningitis. J Clin Invest (2003) 112(5):736–44. 10.1172/JCI17335 12952922 PMC182187

[145] Stoner TD Weston TA Trejo J Doran KS. Group B streptococcal infection and activation of human astrocytes. PLoS One (2015) 10(6):e0128431. 10.1371/journal.pone.0128431 26030618 PMC4452173

[146] Beyrich C Loffler J Kobsar A Speer CP Kneitz S Eigenthaler M. Infection of human coronary artery endothelial cells by group B *Streptococcus* contributes to dysregulation of apoptosis, hemostasis, and innate immune responses. Mediators Inflamm (2011) 2011:971502. 10.1155/2011/971502 21437210 PMC3061215

[147] Puliti M Von Hunolstein C Verwaerde C Bistoni F Orefici G Tissi L. Regulatory role of interleukin-10 in experimental group B streptococcal arthritis. Infect Immun (2002) 70(6):2862–8. 10.1128/IAI.70.6.2862-2868.2002 12010973 PMC128010

[148] Rosati E Fettucciari K Scaringi L Cornacchione P Sabatini R Mezzasoma L Cytokine response to group B *Streptococcus* infection in mice. Scand J Immunol (1998) 47(4):314–23. 10.1046/j.1365-3083.1998.00305.x 9600312

[149] Ernst W Zimara N Hanses F Mannel DN Seelbach-Gobel B Wege AK. Humanized mice, a new model to study the influence of drug treatment on neonatal sepsis. Infect Immun (2013) 81(5):1520–31. 10.1128/IAI.01235-12 23439310 PMC3647987

[150] Teti G Mancuso G Tomasello F. Cytokine appearance and effects of anti-tumor necrosis factor alpha antibodies in a neonatal rat model of group B streptococcal infection. Infect Immun (1993) 61(1):227–35. 8418044 10.1128/iai.61.1.227-235.1993 PMC302709

[151] Biondo C Mancuso G Midiri A Signorino G Domina M Lanza Cariccio V The interleukin-1beta/CXCL1/2/neutrophil axis mediates host protection against group B streptococcal infection. Infect Immun (2014) 82(11):4508–17. 10.1128/IAI.02104-14 25114117 PMC4249330

[152] Biondo C Mancuso G Midiri A Signorino G Domina M Lanza Cariccio V Essential role of interleukin-1 signaling in host defenses against group B *Streptococcus*. MBio (2014) 5(5):e1428–1414. 10.1128/mBio.01428-14 25205091 PMC4166122

[153] Mohammadi N Midiri A Mancuso G Patane F Venza M Venza I Neutrophils directly recognize group B streptococci and contribute to interleukin-1β production during infection. PLoS One (2016) 11(8):e0160249. 10.1371/journal.pone.0160249 27509078 PMC4980021

[154] Cusumano V Mancuso G Genovese F Delfino D Beninati C Losi E Role of gamma interferon in a neonatal mouse model of group B streptococcal disease. Infect Immun (1996) 64(8):2941–4. 8757817 10.1128/iai.64.8.2941-2944.1996 PMC174171

[155] Mancuso G Cusumano V Genovese F Gambuzza M Beninati C Teti G. Role of interleukin 12 in experimental neonatal sepsis caused by group B streptococci. Infect Immun (1997) 65(9):3731–5. 9284145 10.1128/iai.65.9.3731-3735.1997 PMC175532

[156] Cusumano V Midiri A Cusumano VV Bellantoni A De Sossi G Teti G Interleukin-18 is an essential element in host resistance to experimental group B streptococcal disease in neonates. Infect Immun (2004) 72(1):295–300. 10.1128/IAI.72.1.295-300.2004 14688108 PMC344002

[157] Andrade EB Alves J Madureira P Oliveira L Ribeiro A Cordeiro-da-Silva A TLR2-induced IL-10 production impairs neutrophil recruitment to infected tissues during neonatal bacterial sepsis. J Immunol (2013) 191(9):4759–68. 10.4049/jimmunol.1301752 24078699

[158] Madureira P Andrade EB Gama B Oliveira L Moreira S Ribeiro A Inhibition of IL-10 production by maternal antibodies against group B *Streptococcus* GAPDH confers immunity to offspring by favoring neutrophil recruitment. PLoS Pathog (2011) 7(11):e1002363. 10.1371/journal.ppat.1002363 22114550 PMC3219712

[159] Bryan JD Shelver DW. *Streptococcus agalactiae* CspA is a serine protease that inactivates chemokines. J Bacteriol (2009) 191(6):1847–54. 10.1128/JB.01124-08 19114481 PMC2648358

[160] Gupta R Ghosh S Monks B DeOliveira RB Tzeng TC Kalantari P RNA and beta-hemolysin of group B *Streptococcus* induce interleukin-1beta (IL-1beta) by activating NLRP3 inflammasomes in mouse macrophages. J Biol Chem (2014) 289(20):13701–5. 10.1074/jbc.C114.548982 24692555 PMC4022842

[161] Bebien M Hensler ME Davanture S Hsu LC Karin M Park JM The pore-forming toxin beta hemolysin/cytolysin triggers p38 MAPK-dependent IL-10 production in macrophages and inhibits innate immunity. PLoS Pathog (2012) 8(7):e1002812 10.1371/journal.ppat.1002812 22829768 PMC3400567

[162] Madureira P Baptista M Vieira M Magalhaes V Camelo A Oliveira L *Streptococcus agalactiae* GAPDH is a virulence-associated immunomodulatory protein. J Immunol (2007) 178(3):1379–87. 10.4049/jimmunol.178.3.1379 17237385

[163] La Pine TR Joyner JL Augustine NH Kwak SD Hill HR. Defective production of IL-18 and IL-12 by cord blood mononuclear cells influences the T helper-1 interferon gamma response to group B streptococci. Pediatr Res (2003) 54(2):276–81. 10.1203/01.PDR.0000072515.10652.87 12736393

[164] Kwatra G Adrian PV Shiri T Izu A Cutland CL Buchmann EJ Serotype-specific cell-mediated immunity associated with clearance of homotypic group B *Streptococcus* rectovaginal colonization in pregnant women. J Infect Dis (2016) 213(12):1923–6. 10.1093/infdis/jiw056 27029777

[165] Clarke D Letendre C Lecours MP Lemire P Galbas T Thibodeau J Group B *Streptococcus* induces a robust IFN-gamma response by CD4(+) T cells in an in vitro and in vivo model. J Immunol Res (2016) 2016:5290604 10.1155/2016/5290604 26989699 PMC4771917

[166] Smith JM Respess RH Chaffin DG Larsen B Jackman SH. Differences in innate immunologic response to group B *Streptococcus* between colonized and noncolonized women. Infect Dis Obstet Gynecol (2001) 9(3):125–32. 10.1155/S1064744901000230 11516060 PMC1784651

[167] Hordnes K Tynning T Kvam AI Jonsson R Haneberg B. Colonization in the rectum and uterine cervix with group B streptococci may induce specific antibody responses in cervical secretions of pregnant women. Infect Immun (1996) 64(5):1643–52. 8613373 10.1128/iai.64.5.1643-1652.1996 PMC173974

[168] Mitchell K Brou L Bhat G Drobek CO Kramer M Hill A Group B *Streptococcus* colonization and higher maternal IL-1beta concentrations are associated with early term births. J Matern Fetal Neonatal Med (2013) 26(1):56–61. 10.3109/14767058.2012.725789 22946471

[169] Scholl J Nasioudis D Boester A Speleotes M Grunebaum A Witkin SS. Group B *Streptococcus* alters properties of vaginal epithelial cells in pregnant women. Am J Obstet Gynecol (2016) 214(3):383.e1–5. 10.1016/j.ajog.2015.12.053 26928153

[170] Hordnes K Digranes A Haugen IL Helland DE Ulstein M Jonsson R Systemic and mucosal antibody responses to group B streptococci following immunization of the colonic-rectal mucosa. J Reprod Immunol (1995) 28(3):247–62. 10.1016/0165-0378(95)00925-B 7473434

[171] Gendrin C Vornhagen J Ngo L Whidbey C Boldenow E Santana-Ufret V Mast cell degranulation by a hemolytic lipid toxin decreases GBS colonization and infection. Sci Adv (2015) 1(6):e1400225. 10.1126/sciadv.1400225 26425734 PMC4584422

[172] Randis TM Gelber SE Hooven TA Abellar RG Akabas LH Lewis EL Group B *Streptococcus* beta-hemolysin/cytolysin breaches maternal-fetal barriers to cause preterm birth and intrauterine fetal demise in vivo. J Infect Dis (2014) 210(2):265–73. 10.1093/infdis/jiu067 24474814 PMC4092248

[173] Boldenow E Hassan I Chames MC Xi C Loch-Caruso R The trichloroethylene metabolite S-(1,2-dichlorovinyl)-l-cysteine but not trichloroacetate inhibits pathogen-stimulated TNF-alpha in human extraplacental membranes in vitro. Reprod Toxicol (2015) 52:1–6. 10.1016/j.reprotox.2015.01.007 25653212 PMC4426062

[174] Boldenow E Jones S Lieberman RW Chames MC Aronoff DM Xi C Antimicrobial peptide response to group B *Streptococcus* in human extraplacental membranes in culture. Placenta (2013) 34(6):480–5. 10.1016/j.placenta.2013.02.010 23562109 PMC3664555

[175] Zaga-Clavellina V Flores-Espinosa P Pineda-Torres M Sosa-Gonzalez I Vega-Sanchez R Estrada-Gutierrez G Tissue-specific IL-10 secretion profile from term human fetal membranes stimulated with pathogenic microorganisms associated with preterm labor in a two-compartment tissue culture system. J Matern Fetal Neonatal Med (2014) 27(13):1320–7. 10.3109/14767058.2013.857397 24138141

[176] Gravett MG Witkin SS Haluska GJ Edwards JL Cook MJ Novy MJ. An experimental model for intraamniotic infection and preterm labor in rhesus monkeys. Am J Obstet Gynecol (1994) 171(6):1660–7. 10.1016/0002-9378(94)90418-9 7802084

[177] Adams Waldorf KM Gravett MG McAdams RM Paolella LJ Gough GM Carl DJ Choriodecidual group B streptococcal inoculation induces fetal lung injury without intra-amniotic infection and preterm labor in *Macaca nemestrina*. PLoS One (2011) 6(12):e28972. 10.1371/journal.pone.0028972 22216148 PMC3244436

[178] Vanderhoeven JP Bierle CJ Kapur RP McAdams RM Beyer RP Bammler TK Group B streptococcal infection of the choriodecidua induces dysfunction of the cytokeratin network in amniotic epithelium: a pathway to membrane weakening. PLoS Pathog (2014) 10(3):e1003920. 10.1371/journal.ppat.1003920 24603861 PMC3946355

[179] Whidbey C Vornhagen J Gendrin C Boldenow E Samson JM Doering K A streptococcal lipid toxin induces membrane permeabilization and pyroptosis leading to fetal injury. EMBO Mol Med (2015) 7(4):488–505. 10.15252/emmm.201404883 25750210 PMC4403049

[180] Boldenow E Gendrin C Ngo L Bierle C Vornhagen J Coleman M Group B *Streptococcus* circumvents neutrophils and neutrophil extracellular traps during amniotic cavity invasion and preterm labor. Sci Immunol (2016) 1(4):eaah4576. 10.1126/sciimmunol.aah4576 27819066 PMC5089172

[181] Bergeron J Gerges N Guiraut C Grbic D Allard MJ Fortier LC Activation of the IL-1beta/CXCL1/MMP-10 axis in chorioamnionitis induced by inactivated Group B *Streptococcus*. Placenta (2016) 47:116–23. 10.1016/j.placenta.2016.09.016 27780533

[182] Deutscher M Lewis M Zell ER Taylor TH Jr Van Beneden C Schrag S Incidence and severity of invasive *Streptococcus pneumoniae*, group A *Streptococcus*, and group B *Streptococcus* infections among pregnant and postpartum women. Clin Infect Dis (2011) 53(2):114–23. 10.1093/cid/cir325 21690617

[183] Bergeron JD Deslauriers J Grignon S Fortier LC Lepage M Stroh T White matter injury and autistic-like behavior predominantly affecting male rat offspring exposed to group B streptococcal maternal inflammation. Dev Neurosci (2013) 35(6):504–15. 10.1159/000355656 24246964

[184] Allard MJ Bergeron JD Baharnoori M Srivastava LK Fortier LC Poyart C A sexually dichotomous, autistic-like phenotype is induced by group B *Streptococcus* maternofetal immune activation. Autism Res (2017) 10(2):233–45. 10.1002/aur.1647 27220806

[185] Jensen NE Andersen BL. The prevalence of group B streptococci in human urogenital secretions. Scand J Infect Dis (1979) 11(3):199–202. 10.3109/inf.1979.11.issue-3.04 118523

[186] Honig E Mouton JW van der Meijden WI. Can group B streptococci cause symptomatic vaginitis? Infect Dis Obstet Gynecol (1999) 7(4):206–9. 10.1155/S1064744999000368 10449271 PMC1784743

[187] Feikin DR Thorsen P Zywicki S Arpi M Westergaard JG Schuchat A. Association between colonization with group B streptococci during pregnancy and preterm delivery among Danish women. Am J Obstet Gynecol (2001) 184(3):427–33. 10.1067/mob.2001.109936 11228498

[188] Regan JA Klebanoff MA Nugent RP Eschenbach DA Blackwelder WC Lou Y Colonization with group B streptococci in pregnancy and adverse outcome. VIP Study Group. Am J Obstet Gynecol (1996) 174(4):1354–60. 10.1016/S0002-9378(96)70684-1 8623869

[189] Regan JA Chao S James LS. Premature rupture of membranes, preterm delivery, and group B streptococcal colonization of mothers. Am J Obstet Gynecol (1981) 141(2):184–6. 10.1016/S0002-9378(16)32589-3 7025636

[190] Matorras R Garcia Perea A Omenaca F Usandizaga JA Nieto A Herruzo R. Group B *Streptococcus* and premature rupture of membranes and preterm delivery. Gynecol Obstet Invest (1989) 27(1):14–8. 10.1159/000293607 2646185

[191] Valkenburg-van den Berg AW Sprij AJ Dekker FW Dorr PJ Kanhai HH. Association between colonization with group B *Streptococcus* and preterm delivery: a systematic review. Acta Obstet Gynecol Scand (2009) 88(9):958–67. 10.1080/00016340903176800 19657755

[192] Muller AE Oostvogel PM Steegers EA Dorr PJ. Morbidity related to maternal group B streptococcal infections. Acta Obstet Gynecol Scand (2006) 85(9):1027–37. 10.1080/00016340600780508 16929406

[193] Yancey MK Duff P Clark P Kurtzer T Frentzen BH Kubilis P. Peripartum infection associated with vaginal group B streptococcal colonization. Obstet Gynecol (1994) 84(5):816–9. 7936518

[194] Krohn MA Hillier SL Baker CJ. Maternal peripartum complications associated with vaginal group B streptococci colonization. J Infect Dis (1999) 179(6):1410–5. 10.1086/314756 10228062

[195] Nan C Dangor Z Cutland CL Edwards MS Madhi SA Cunnington MC. Maternal group B *Streptococcus*-related stillbirth: a systematic review. BJOG (2015) 122(11):1437–45. 10.1111/1471-0528.13527 26177561

[196] Prince AL Ma J Kannan PS Alvarez M Gisslen T Harris RA The placental membrane microbiome is altered among subjects with spontaneous preterm birth with and without chorioamnionitis. Am J Obstet Gynecol (2016) 214(5):627.e1–16. 10.1016/j.ajog.2016.01.193 26965447 PMC4909356

[197] Vornhagen J Quach P Boldenow E Merillat S Whidbey C Ngo LY Bacterial hyaluronidase promotes ascending GBS infection and preterm birth. MBio (2016) 7(3):e00781–16. 10.1128/mBio.00781-16 27353757 PMC4937215

[198] Kline KA Lewis AL. Gram-positive uropathogens, polymicrobial urinary tract infection, and the emerging microbiota of the urinary tract. Microbiol Spectr (2016) 4(2):UTI-0012–2012. 10.1128/microbiolspec.UTI-0012-2012 27227294 PMC4888879

[199] Ulett KB Benjamin WH Jr Zhuo F Xiao M Kong F Gilbert GL Diversity of group B *Streptococcus* serotypes causing urinary tract infection in adults. J Clin Microbiol (2009) 47(7):2055–60. 10.1128/JCM.00154-09 19439533 PMC2708523

[200] Wood EG Dillon HC Jr. A prospective study of group B streptococcal bacteriuria in pregnancy. Am J Obstet Gynecol (1981) 140(5):515–20. 10.1016/0002-9378(81)90226-X 7018248

[201] Hill JB Sheffield JS McIntire DD Wendel GD Jr. Acute pyelonephritis in pregnancy. Obstet Gynecol (2005) 105(1):18–23. 10.1097/01.AOG.0000149154.96285.a0 15625136

[202] Kessous R Weintraub AY Sergienko R Lazer T Press F Wiznitzer A Bacteruria with group-B *Streptococcus*: is it a risk factor for adverse pregnancy outcomes? J Matern Fetal Neonatal Med (2012) 25(10):1983–6. 10.3109/14767058.2012.671872 22530608

[203] Perez-Moreno MO Pico-Plana E Grande-Armas J Centelles-Serrano MJ Arasa-Subero M Ochoa NC Group B streptococcal bacteriuria during pregnancy as a risk factor for maternal intrapartum colonization: a prospective cohort study. J Med Microbiol (2017) 66(4):454–60. 10.1099/jmm.0.000465 28463661

[204] Haider G Zehra N Munir AA Haider A. Risk factors of urinary tract infection in pregnancy. J Pak Med Assoc (2010) 60(3):213–6. 20225781

[205] Kline KA Schwartz DJ Gilbert NM Lewis AL. Impact of host age and parity on susceptibility to severe urinary tract infection in a murine model. PLoS One (2014) 9(5):e97798. 10.1371/journal.pone.0097798 24835885 PMC4024022

[206] Kulkarni R Randis TM Antala S Wang A Amaral FE Ratner AJ. β-Hemolysin/cytolysin of group B *Streptococcus* enhances host inflammation but is dispensable for establishment of urinary tract infection. PLoS One (2013) 8(3):e59091. 10.1371/journal.pone.0059091 23505569 PMC3591438

[207] Leclercq SY Sullivan MJ Ipe DS Smith JP Cripps AW Ulett GC Pathogenesis of *Streptococcus* urinary tract infection depends on bacterial strain and beta-hemolysin/cytolysin that mediates cytotoxicity, cytokine synthesis, inflammation and virulence. Sci Rep (2016) 6:29000 10.1038/srep29000 27383371 PMC4935997

[208] Kubin V Mrastikova H Paulova M Motlova J Franek J. Group B streptococci in the milk of lactating mothers. Zentralbl Bakteriol Mikrobiol Hyg A (1987) 265(1–2):210–7. 3314258 10.1016/s0176-6724(87)80168-2

[209] Kvist LJ Larsson BW Hall-Lord ML Steen A Schalen C. The role of bacteria in lactational mastitis and some considerations of the use of antibiotic treatment. Int Breastfeed J (2008) 3:6. 10.1186/1746-4358-3-6 18394188 PMC2322959

[210] Andreas NJ Al-Khalidi A Jaiteh M Clarke E Hyde MJ Modi N Role of human milk oligosaccharides in group B *Streptococcus* colonisation. Clin Transl Immunology (2016) 5(8):e99. 10.1038/cti.2016.43 27588204 PMC5007626

[211] Le Doare K Kampmann B. Breast milk and group B streptococcal infection: vector of transmission or vehicle for protection? Vaccine (2014) 32(26):3128–32. 10.1016/j.vaccine.2014.04.020 24736004 PMC4037808

[212] Zimmermann P Gwee A Curtis N. The controversial role of breast milk in GBS late-onset disease. J Infect (2017) 74(Suppl 1):S34–40. 10.1016/S0163-4453(17)30189-5 28646960

[213] Boggess KA Watts DH Hillier SL Krohn MA Benedetti TJ Eschenbach DA. Bacteremia shortly after placental separation during cesarean delivery. Obstet Gynecol (1996) 87(5 Pt 1):779–84. 10.1016/0029-7844(96)00037-3 8677085

[214] Hall RT Barnes W Krishnan L Harris DJ Rhodes PG Fayez J Antibiotic treatment of parturient women colonized with group B streptococci. Am J Obstet Gynecol (1976) 124(6):630–4. 10.1016/0002-9378(76)90065-X 769556

[215] Gardner SE Yow MD Leeds LJ Thompson PK Mason EO Jr Clark DJ. Failure of penicillin to eradicate group B streptococcal colonization in the pregnant woman. A couple study. Am J Obstet Gynecol (1979) 135(8):1062–5. 10.1016/0002-9378(79)90737-3 391044

[216] Szymusik I Kosinska-Kaczynska K Krolik A Skurnowicz M Pietrzak B Wielgos M. The usefulness of the universal culture-based screening and the efficacy of intrapartum prophylaxis of group B *Streptococcus* infection. J Matern Fetal Neonatal Med (2014) 27(9):968–70. 10.3109/14767058.2013.845659 24047083

[217] Gilbert GL Hewitt MC Turner CM Leeder SR. Compliance with protocols for prevention of neonatal group B streptococcal sepsis: practicalities and limitations. Infect Dis Obstet Gynecol (2003) 11(1):1–9. 10.1155/S1064744903000012 12839627 PMC1852267

[218] Scasso S Laufer J Rodriguez G Alonso JG Sosa CG. Vaginal group B *Streptococcus* status during intrapartum antibiotic prophylaxis. Int J Gynaecol Obstet (2015) 129(1):9–12. 10.1016/j.ijgo.2014.10.018 25577036

[219] Fairlie T Zell ER Schrag S. Effectiveness of intrapartum antibiotic prophylaxis for prevention of early-onset group B streptococcal disease. Obstet Gynecol (2013) 121(3):570–7. 10.1097/AOG.0b013e318280d4f6 23635620

[220] Turrentine M. Intrapartum antibiotic prophylaxis for group B *Streptococcus*: has the time come to wait more than 4 hours? Am J Obstet Gynecol (2014) 211(1):15–7. 10.1016/j.ajog.2013.12.010 24315859

[221] Berardi A Rossi C Creti R China M Gherardi G Venturelli C Group B streptococcal colonization in 160 mother-baby pairs: a prospective cohort study. J Pediatr (2013) 163(4):1099–104.e1. 10.1016/j.jpeds.2013.05.064 23866714

[222] Toyofuku M Morozumi M Hida M Satoh Y Sakata H Shiro H Effects of intrapartum antibiotic prophylaxis on neonatal acquisition of group B streptococci. J Pediatr (2017) 190:169–73.e1. 10.1016/j.jpeds.2017.07.039 29144242

[223] Spaetgens R DeBella K Ma D Robertson S Mucenski M Davies HD. Perinatal antibiotic usage and changes in colonization and resistance rates of group B *Streptococcus* and other pathogens. Obstet Gynecol (2002) 100(3):525–33. 10.1097/00006250-200209000-00020 12220773

[224] Stoll BJ Hansen NI Sanchez PJ Faix RG Poindexter BB Van Meurs KP Early onset neonatal sepsis: the burden of group B streptococcal and *E. coli* disease continues. Pediatrics (2011) 127(5):817–26. 10.1542/peds.2010-2217 21518717 PMC3081183

[225] Weston EJ Pondo T Lewis MM Martell-Cleary P Morin C Jewell B The burden of invasive early-onset neonatal sepsis in the United States, 2005–2008. Pediatr Infect Dis J (2011) 30(11):937–41. 10.1097/INF.0b013e318223bad2 21654548 PMC3193564

[226] Bauserman MS Laughon MM Hornik CP Smith PB Benjamin DK Jr Clark RH Group B *Streptococcus* and *Escherichia coli* infections in the intensive care nursery in the era of intrapartum antibiotic prophylaxis. Pediatr Infect Dis J (2013) 32(3):208–12. 10.1097/INF.0b013e318275058a 23011013 PMC3572304

[227] Centers for Disease Control and Prevention (CDC). Active Bacterial Core Surveillance Report, Emerging Infections Program Network, Group B Streptococcus, 2014. (2014). Report No gbs14. Centers for Disease Control and Prevention.

[228] Levine EM Ghai V Barton JJ Strom CM. Intrapartum antibiotic prophylaxis increases the incidence of Gram-negative neonatal sepsis. Infect Dis Obstet Gynecol (1999) 7(4):210–3. 10.1155/S106474499900037X 10449272 PMC1784737

[229] Terrone DA Rinehart BK Einstein MH Britt LB Martin JN Jr Perry KG. Neonatal sepsis and death caused by resistant *Escherichia coli*: possible consequences of extended maternal ampicillin administration. Am J Obstet Gynecol (1999) 180(6 Pt 1):1345–8. 10.1016/S0002-9378(99)70017-7 10368469

[230] Ecker KL Donohue PK Kim KS Shepard JA Aucott SW. The impact of group B *Streptococcus* prophylaxis on early onset neonatal infections. J Neonatal Perinatal Med (2013) 6(1):37–44. 10.3233/NPM-1363312 24246457

[231] Keski-Nisula L Kyynarainen HR Karkkainen U Karhukorpi J Heinonen S Pekkanen J. Maternal intrapartum antibiotics and decreased vertical transmission of *Lactobacillus* to neonates during birth. Acta Paediatr (2013) 102(5):480–5. 10.1111/apa.12186 23398392

[232] Aloisio I Mazzola G Corvaglia LT Tonti G Faldella G Biavati B Influence of intrapartum antibiotic prophylaxis against group B *Streptococcus* on the early newborn gut composition and evaluation of the anti-*Streptococcus* activity of *Bifidobacterium* strains. Appl Microbiol Biotechnol (2014) 98(13):6051–60. 10.1007/s00253-014-5712-9 24687755

[233] Corvaglia L Tonti G Martini S Aceti A Mazzola G Aloisio I Influence of intrapartum antibiotic prophylaxis for group B *Streptococcus* on gut microbiota in the first month of life. J Pediatr Gastroenterol Nutr (2016) 62(2):304–8. 10.1097/MPG.0000000000000928 26237371

[234] Azad MB Konya T Persaud RR Guttman DS Chari RS Field CJ Impact of maternal intrapartum antibiotics, method of birth and breastfeeding on gut microbiota during the first year of life: a prospective cohort study. BJOG (2016) 123(6):983–93. 10.1111/1471-0528.13601 26412384

[235] Cassidy-Bushrow AE Sitarik A Levin AM Lynch SV Havstad S Ownby DR Maternal group B *Streptococcus* and the infant gut microbiota. J Dev Orig Health Dis (2016) 7(1):45–53. 10.1017/S2040174415001361 26264560 PMC4847528

[236] Dinsmoor MJ Viloria R Lief L Elder S. Use of intrapartum antibiotics and the incidence of postnatal maternal and neonatal yeast infections. Obstet Gynecol (2005) 106(1):19–22. 10.1097/01.AOG.0000164049.12159.bd 15994612

[237] Roesch LF Silveira RC Corso AL Dobbler PT Mai V Rojas BS Diversity and composition of vaginal microbiota of pregnant women at risk for transmitting group B *Streptococcus* treated with intrapartum penicillin. PLoS One (2017) 12(2):e0169916. 10.1371/journal.pone.0169916 28178310 PMC5298327

[238] Kenyon S Pike K Jones DR Brocklehurst P Marlow N Salt A Childhood outcomes after prescription of antibiotics to pregnant women with spontaneous preterm labour: 7-year follow-up of the ORACLE II trial. Lancet (2008) 372(9646):1319–27. 10.1016/S0140-6736(08)61203-9 18804276

[239] Saari A Virta LJ Sankilampi U Dunkel L Saxen H. Antibiotic exposure in infancy and risk of being overweight in the first 24 months of life. Pediatrics (2015) 135(4):617–26. 10.1542/peds.2014-3407 25825533

[240] Mueller NT Whyatt R Hoepner L Oberfield S Dominguez-Bello MG Widen EM Prenatal exposure to antibiotics, cesarean section and risk of childhood obesity. Int J Obes (Lond) (2015) 39(4):665–70. 10.1038/ijo.2014.180 25298276 PMC4390478

[241] McCloskey K Vuillermin P Carlin JB Cheung M Skilton MR Tang ML Perinatal microbial exposure may influence aortic intima-media thickness in early infancy. Int J Epidemiol (2017) 46(1):209–18. 10.1093/ije/dyw042 27059546

[242] Chu S Yu H Chen Y Chen Q Wang B Zhang J. Periconceptional and gestational exposure to antibiotics and childhood asthma. PLoS One (2015) 10(10):e0140443. 10.1371/journal.pone.0140443 26488397 PMC4619063

[243] Bjorksten B Sepp E Julge K Voor T Mikelsaar M. Allergy development and the intestinal microflora during the first year of life. J Allergy Clin Immunol (2001) 108(4):516–20. 10.1067/mai.2001.118130 11590374

[244] Dowhower Karpa K Paul IM Leckie JA Shung S Carkaci-Salli N Vrana KE A retrospective chart review to identify perinatal factors associated with food allergies. Nutr J (2012) 11:87. 10.1186/1475-2891-11-87 23078601 PMC3493351

[245] Seedat F Stinton C Patterson J Geppert J Tan B Robinson ER Adverse events in women and children who have received intrapartum antibiotic prophylaxis treatment: a systematic review. BMC Pregnancy Childbirth (2017) 17(1):247. 10.1186/s12884-017-1432-3 28747160 PMC5530570

[246] Schrag SJ Verani JR. Intrapartum antibiotic prophylaxis for the prevention of perinatal group B streptococcal disease: experience in the United States and implications for a potential group B streptococcal vaccine. Vaccine (2013) 31(Suppl 4):D20–6. 10.1016/j.vaccine.2012.11.056 23219695 PMC11843781

[247] Ohlsson A Shah VS Intrapartum antibiotics for known maternal group B streptococcal colonization. Cochrane Database Syst Rev (2014) 6:CD007467 10.1002/14651858.CD007467.pub4 24915629

[248] Baker CJ Kasper DL. Group B streptococcal vaccines. Rev Infect Dis (1985) 7(4):458–67. 10.1093/clinids/7.4.458 3898306

[249] Brzychczy-Wloch M Gorska S Brzozowska E Gamian A Heczko PB Bulanda M. Identification of high immunoreactive proteins from *Streptococcus agalactiae* isolates recognized by human serum antibodies. FEMS Microbiol Lett (2013) 349(1):61–70. 10.1111/1574-6968.12292 24152143

[250] Lin FY Weisman LE Azimi PH Philips JB III Clark P Regan J Level of maternal IgG anti-group B *Streptococcus* type III antibody correlated with protection of neonates against early-onset disease caused by this pathogen. J Infect Dis (2004) 190(5):928–34. 10.1086/422756 15295698

[251] Baker JA Lewis EL Byland LM Bonakdar M Randis TM Ratner AJ. Mucosal vaccination promotes clearance of *Streptococcus agalactiae* vaginal colonization. Vaccine (2017) 35(9):1273–80. 10.1016/j.vaccine.2017.01.029 28162823 PMC5319564

[252] Edwards MS Hall MA Rench MA Baker CJ. Patterns of immune response among survivors of group B streptococcal meningitis. J Infect Dis (1990) 161(1):65–70. 10.1093/infdis/161.1.65 2404076

[253] Baker CJ. The spectrum of perinatal group B streptococcal disease. Vaccine (2013) 31(Suppl 4):D3–6. 10.1016/j.vaccine.2013.02.030 23973344

[254] Madhi SA Dangor Z Heath PT Schrag S Izu A Sobanjo-Ter Meulen A Considerations for a phase-III trial to evaluate a group B *Streptococcus* polysaccharide-protein conjugate vaccine in pregnant women for the prevention of early- and late-onset invasive disease in young-infants. Vaccine (2013) 31(Suppl 4):D52–7. 10.1016/j.vaccine.2013.02.029 23973347

[255] Madhi SA Koen A Cutland CL Jose L Govender N Wittke F Antibody kinetics and response to routine vaccinations ininfants born to women who received an investigational trivalent group B *Streptococcus* polysaccharide CRM197-conjugate vaccine during pregnancy. Clin Infect Dis (2017) 65(11):1897–904. 10.1093/cid/cix666 29029127 PMC5848233

[256] Le Doare K Faal A Jaiteh M Sarfo F Taylor S Warburton F Association between functional antibody against group B *Streptococcus* and maternal and infant colonization in a Gambian cohort. Vaccine (2017) 35(22):2970–8. 10.1016/j.vaccine.2017.04.013 28449969 PMC5432431

[257] Springman AC Lacher DW Waymire EA Wengert SL Singh P Zadoks RN Pilus distribution among lineages of group B *Streptococcus*: an evolutionary and clinical perspective. BMC Microbiol (2014) 14:159. 10.1186/1471-2180-14-159 24943359 PMC4074840

[258] Lin SM Jang AY Zhi Y Gao S Lim S Lim JH Immunization with a latch peptide provides serotype-independent protection against group B *Streptococcus* infection in mice. J Infect Dis (2017) 217(1):93–102. 10.1093/infdis/jix565 29106586 PMC5854025

[259] Li J Kasper DL Ausubel FM Rosner B Michel JL. Inactivation of the alpha C protein antigen gene, *bca*, by a novel shuttle/suicide vector results in attenuation of virulence and immunity in group B *Streptococcus*. Proc Natl Acad Sci U S A (1997) 94(24):13251–6. 10.1073/pnas.94.24.13251 9371832 PMC24295

[260] Xue G Yu L Li S Shen X. Intranasal immunization with GBS surface protein Sip and ScpB induces specific mucosal and systemic immune responses in mice. FEMS Immunol Med Microbiol (2010) 58(2):202–10. 10.1111/j.1574-695X.2009.00623.x 19912341

[261] Oster G Edelsberg J Hennegan K Lewin C Narasimhan V Slobod K Prevention of group B streptococcal disease in the first 3 months of life: would routine maternal immunization during pregnancy be cost-effective? Vaccine (2014) 32(37):4778–85. 10.1016/j.vaccine.2014.06.003 24992717

[262] Kim SY Nguyen C Russell LB Tomczyk S Abdul-Hakeem F Schrag SJ Cost-effectiveness of a potential group B streptococcal vaccine for pregnant women in the United States. Vaccine (2017) 35(45):6238–47. 10.1016/j.vaccine.2017.08.085 28951085

[263] Kobayashi M Vekemans J Baker CJ Ratner AJ Le Doare K Schrag SJ. Group B *Streptococcus* vaccine development: present status and future considerations, with emphasis on perspectives for low and middle income countries. F1000Res (2016) 5:2355. 10.12688/f1000research.9363.1 27803803 PMC5070600

[264] Madhi SA Dangor Z. Prospects for preventing infant invasive GBS disease through maternal vaccination. Vaccine (2017) 35(35 Pt A):4457–60. 10.1016/j.vaccine.2017.02.025 28237500

[265] Abachi S Lee S Rupasinghe HP. Molecular mechanisms of inhibition of *Streptococcus* species by phytochemicals. Molecules (2016) 21(2):215. 10.3390/molecules21020215 26901172 PMC6273676

[266] Dhouioui M Boulila A Jemli M Schiets F Casabianca H Zina MS Fatty acids composition and antibacterial activity of *Aristolochia longa* L. and *Bryonia dioica* Jacq. growing wild in Tunisia. J Oleo Sci (2016) 65(8):655–61. 10.5650/jos.ess16001 27430383

[267] Moncla BJ Pryke K Isaacs CE. Killing of *Neisseria gonorrhoeae, Streptococcus agalactiae* (group B *Streptococcus*), *Haemophilus ducreyi*, and vaginal *Lactobacillus* by 3-O-octyl-sn-glycerol. Antimicrob Agents Chemother (2008) 52(4):1577–9. 10.1128/AAC.01023-07 18227178 PMC2292550

[268] Ardolino LI Meloni M Brugali G Corsini E Galli CL. Preclinical evaluation of tolerability of a selective, bacteriostatic, locally active vaginal formulation. Curr Ther Res Clin Exp (2016) 83:13–21. 10.1016/j.curtheres.2016.07.002 27766122 PMC5067097

[269] Cavaco CK Patras KA Zlamal JE Thoman ML Morgan EL Sanderson SD A novel C5a-derived immunobiotic peptide reduces *Streptococcus agalactiae* colonization through targeted bacterial killing. Antimicrob Agents Chemother (2013) 57(11):5492–9. 10.1128/AAC.01590-13 23979760 PMC3811312

[270] Ohlsson A Shah VS Stade BC Vaginal chlorhexidine during labour to prevent early-onset neonatal group B streptococcal infection. Cochrane Database Syst Rev (2014) 12:CD003520 10.1002/14651858.CD003520.pub3 PMC11262555 25504106

[271] Falagas M Betsi GI Athanasiou S. Probiotics for the treatment of women with bacterial vaginosis. Clin Microbiol Infect (2007) 13(7):657–64. 10.1111/j.1469-0691.2007.01688.x 17633390

[272] Homayouni A Bastani P Ziyadi S Mohammad-Alizadeh-Charandabi S Ghalibaf M Mortazavian AM Effects of probiotics on the recurrence of bacterial vaginosis: a review. J Low Genit Tract Dis (2014) 18(1):79–86. 10.1097/LGT.0b013e31829156ec 24299970

[273] Acikgoz ZC Gamberzade S Gocer S Ceylan P. [Inhibitor effect of vaginal lactobacilli on group B streptococci]. Mikrobiyol Bul (2005) 39(1):17–23. 15900833

[274] Bodaszewska M Brzychczy-Wloch M Gosiewski T Adamski P Strus M Heczko PB. [Evaluation of group B *Streptococcus* susceptibility to lactic acid bacteria strains]. Med Dosw Mikrobiol (2010) 62(2):153–61. 20873488

[275] Ruiz FO Gerbaldo G Garcia MJ Giordano W Pascual L Barberis IL. Synergistic effect between two bacteriocin-like inhibitory substances produced by *Lactobacilli* strains with inhibitory activity for *Streptococcus agalactiae*. Curr Microbiol (2012) 64(4):349–56. 10.1007/s00284-011-0077-0 22231454

[276] Bodaszewska-Lubas M Brzychczy-Wloch M Gosiewski T Heczko PB Antibacterial activity of selected standard strains of lactic acid bacteria producing bacteriocins – pilot study. Postepy Hig Med Dosw (Online) (2012) 66:787–94. 10.5604/17322693.1015531 23175332

[277] Juarez Tomas MS Saralegui Duhart CI De Gregorio PR Vera Pingitore E Nader-Macias ME. Urogenital pathogen inhibition and compatibility between vaginal *Lactobacillus* strains to be considered as probiotic candidates. Eur J Obstet Gynecol Reprod Biol (2011) 159(2):399–406. 10.1016/j.ejogrb.2011.07.010 21862199

[278] Ho M Chang YY Chang WC Lin HC Wang MH Lin WC Oral *Lactobacillus rhamnosus* GR-1 and *Lactobacillus reuteri* RC-14 to reduce group B *Streptococcus* colonization in pregnant women: a randomized controlled trial. Taiwan J Obstet Gynecol (2016) 55(4):515–8. 10.1016/j.tjog.2016.06.003 27590374

[279] Hanson L Vandevusse L Duster M Warrack S Safdar N. Feasibility of oral prenatal probiotics against maternal group B *Streptococcus* vaginal and rectal colonization. J Obstet Gynecol Neonatal Nurs (2014) 43(3):294–304. 10.1111/1552-6909.12308 24754328

[280] Patras KA Wescombe PA Rosler B Hale JD Tagg JR Doran KS. *Streptococcus salivarius* K12 limits group B *Streptococcus* vaginal colonization. Infect Immun (2015) 83(9):3438–44. 10.1128/IAI.00409-15 26077762 PMC4534663

[281] Patras KA Analyzing Group B Streptococcal and Host Factors Influencing Vaginal Colonization and Exploring Therapeutic Interventions. San Diego, CA: University of California San Diego (2015).

